# Studies of the Dissemination and Quantitative Transplantation of a Lymphocytic Leukaemia of CBA Mice

**DOI:** 10.1038/bjc.1958.47

**Published:** 1958-09

**Authors:** H. B. Hewitt

## Abstract

**Images:**


### STUDIES OF THE DISSEMINATION AND QUANTITATIVE TRANSPLANTATION OF A LYMPHOCYTIC LEUKAEMIA OF CBA MICE

H. B. HEWITT

*From the John Burford Carlill Laboratories, Westminster Hospital, London*

Received for publication May 27 1958

THIS paper reports various transplantation studies of a lymphocytic type of leukaemia of CBA mice, the condition having originated spontaneously. The object of these studies has been to provide a background of general information concerning the natural history of the transplanted disease, and thus to improve the interpretation of radiobiological experiments aimed at eradication of the disease. Such features as the rate of migration of the leukaemia cells from the site of injection, the number of cells required for successful transplantation of the condition, the possiblity of cell-free transfer, and the latent periods elapsing between the injection of cells and death of the mice, assume considerable importance in relation to the stage at which an animal is subjected to experimental therapeutic procedures (Goldin, Venditti, Humphreys, Dennis, Mantel and Greenhouse, 1954 ; Skipper, Schabel, Bell, Thomson and Johnson, 1957).

All the experiments to be described were done with material from the 4th to the 74th passages. Although it would have been desirable to have confined many of the experiments within a shorter period of the leukaemia's history, such a course was prohibited by limitation of the rate of supply of mice for experiment. It can be stated here, however, that no change in the character of the disease has been observed over the period in which the experiments were done. The mean latent period between injection of cells and death of the injected passage mice, which would have been expected to fall with increase in virulence, has shown no progressive change.

### MATERIALS AND METHODS

*Mice.*—CBA mice bred in this laboratory by brother-to-sister mating and of the same colony as that in which the leukaemia arose, were used in the majority of experiments ; unless otherwise stated, the mice were 2–6 months old when used in experiments. For the few homologous transplantation experiments albino mice of a uniform heterozygous strain were used ; these also were bred in this laboratory by brother-to-sister mating.

*Preparation of leukaemia cell suspensions.*—Routine serial passages of the leukaemia were made by injecting intraperitoneally 0·2 ml. of the supernatant fluid of a 10 per cent suspension in Tyrode solution of minced leukaemic liver which had been allowed to stand for about 2 minutes ; each inoculum contained about $10^7$ morphologically intact leukaemia cells. Single-cell suspensions for quantitative transplantation studies were prepared as follows : fresh leukaemic liver was finely minced with scissors and suspended in about nineteen times its volume of

5 per cent CBA serum (stored at — 20° C.) in Tyrode solution. After standing at 2° C. in a 4 in. × ½ in. tube for 5 minutes to allow the gross tissue lumps to settle, the upper three-quarters of the overlying fluid was removed and used to fill one or more Kahn tubes up to a level 6 cm. above the bottom of the tube. The Kahn tubes, fitted with rubber bungs, were allowed to stand upright for one hour at 0–2° C. The upper 2 cm. portions of the columns of fluid were then carefully removed with a fine Pasteur pipette, pooled and mixed. Samples of suspensions so made have been found consistently to be free from clumps of leukaemia cells. It may be mentioned here that similarly prepared suspensions from normal liver (as distinct from spleen, lymph gland, etc.) were almost free from single nucleated cells ; only occasional macrophages were seen in such suspensions. Thus, it appeared likely that an overwhelming majority of the single cells released from infiltrated liver were malignant cells.

*Cell counts.*—Single-cell suspensions were counted in a counting chamber by phase-contrast microscopy. The predominant cell in leukaemic liver preparations, believed to be the viable leukaemia cell, was a non-granular spherical cell about 7·5 $\mu$ in diameter, of a yellowish tint, with a halo, and showing no clear-cut distinction between nucleus and cytoplasm. Normal cells present included erythrocytes, macrophages, polymorphonuclear leucocytes and lymphocytes. Only the lymphocytes presented difficulty in their distinction from the malignant cells. Comparison with lymphocytes from normal lymph glands, examined under similar conditions, showed that the lymphocytes were slightly smaller and more grey in tint than the malignant cells. It must be admitted, however, that a small error would have been introduced by failure to distinguish a lymphocyte from a small malignant cell. Occasional degenerate malignant cells were seen ; these were either partially plasmolysed cells with a clear-cut nuclear membrane, or very small circular or pyriform cells having a very distinct condensed nucleus. The count was expressed as the density per 0·2 ml. of apparently viable malignant cells present in the initial suspension.

*Titration of leukaemia cells.*—Serial five- or tenfold dilutions in the serum-Tyrode medium were made of counted single-cell suspensions prepared as described above. 0·2 ml volumes of each of a series of 4–6 such dilutions were injected into groups of 5–10 mice. The mean number of leukaemia cells injected into mice of any group was calculated by multiplying the cell count of the starting suspension by the dilution factor. The injected mice were observed for a total period of 90 days, during which the mortality for each group was recorded. All mice dying were inspected for gross signs of leukaemia, mice dying of other causes being excluded from the data. The 90-day period of observation was at least twice the longest latent period observed in any mouse developing leukaemia after injection with leukaemia cells. From the data, consisting of the mortalities associated with the mean cell doses injected, the number of cells required to convey leukaemia to half a group of injected mice (the TD50) was calculated by an accumulation method (Reed and Muench, 1938). The range of cell doses injected in any experiment was selected in relation to the predicted TD50.

*Preparation of cell-free extracts of leukaemic tissues.*—Minced leukaemic tissue was ground in a mortar with sand to give a 10–20 per cent suspension in serum-Tyrode medium ; the suspension was centrifuged at 3000–4000 r.p.m. for 10 minutes and the upper three-quarters of the supernatant fluid was recentrifuged similarly ; the upper half of the final centrifugate constituted the cell-free extract.

*Blood smears and organ impression smears.*—Smears were dried in air, fixed with methanol and stained for 7 minutes with a 1 : 1 mixture of Giemsa stain and buffer (pH 6·8).

*Histology.*—Tissues were fixed in Bouin's fluid and sections were stained with haematoxylin and eosin.

*Injections.*—Mice were injected subcutaneously over the dorsal region, intraperitoneally or intravenously, using a tuberculin syringe carrying a Schick-test needle. Leukaemia cells were usually injected in a volume of 0·2 ml.

All animal work was carried out using a fully aseptic technique. In the quantitative studies the leukaemia cell suspensions were kept at 0–2° C. throughout the entire period from their liberation from the leukaemic organ until their injection. The cells of counted suspensions were maintained in uniform suspension, until their injection, by intermittent mixing.

### OBSERVATIONS AND EXPERIMENTS

### *Origin of the leukaemia*

The leukaemia was first observed in an untreated 6-month-old male CBA mouse of the same colony as that providing the mice for transplantation experiments. No other leukaemic mouse has been found in the colony during the four years since its inception. The peripheral blood of the sick mouse showed anaemia (haemoglobin 60 per cent of normal) and leucocytosis (40,000/c.mm.); polymorphonuclear leucocytes were very few and the predominant circulating leucocyte was a fairly large diffuse mononuclear cell resembling a lymphoblast; there was marked anisocytosis and poikilocytosis among the erythrocytes and there were numerous macrocytes. The lymph nodes of all groups examined were enlarged to about 4–6 mm. in their longest diameter. The spleen, liver and kidneys were obviously enlarged. Histological sections of lymph glands, spleen, liver and kidneys showed heavy infiltration with the malignant cells; in spleen and lymph gland, normal lymphocytes appeared to have been largely replaced by the malignant cells; infiltration of the liver and kidney had taken place without any apparent deterioration of the health of the normal epithelial components of these tissues.

These findings were of particular interest because they included features which are not normally seen in the passage mice. These features were as follows: the erythrocyte abnormalities, the absence of a polymorphonuclear leucocytosis in the presence of a high circulating malignant cell count, and the gross enlargement of lymph nodes. The possible significance of these peculiarities of the original mouse with spontaneous leukaemia will be discussed later.

### *Description of the transplanted leukaemic condition*

Between 9 and 11 days after the intraperitoneal injection of several million leukaemia cells, there is a sharp decline in the food consumption of injected mice, so that by the 11th day the average consumption of food is less than one-sixth

---

EXPLANATION OF PLATE

FIG. 1.—Infiltrated liver of moribund leukaemic mouse. H. and E. × 200.

FIG. 2.—Degeneration of a zone of cerebellum in a leukaemic mouse showing neurological signs. H. and E. × 54.

1

2

Hewitt.

of that of uninjected control mice. From the 9th day onward there is a progressive decline in the average body weight of the injected mice. The first sign of sickness, reduced activity, appears by the 11th day; sickness rapidly increases, and all the mice of an injected group are dead or moribund by the 15th day. One quarter of all the mice injected, with whatever cell dose, develop gross signs of disturbance of the central nervous system within 2 days of death; these signs, referable to dysfunction of the vestibular apparatus or cerebellum, consist of "rolling", crawling in circles, ataxia, asymmetrical posturing of the head, and "whipping" of the tail. These signs are increased by disturbance of the affected mice and appear also to be aggravated by attempts of the mouse to initiate voluntary movements.

Dissection of mice with advanced leukaemia showed: slight enlargement of the lymph glands, often with periglandular hyperaemia; considerable enlargement of the liver and spleen, both of which were paler than normal; enlargement of the kidneys, which were mottled with pale areas; occasionally, a haemorrhagic condition of one or more lobes of the lung; and, in some cases, the appearance of dense white streaks on the epicardium, which appear to follow the direction of the superficial ventricular muscle fibres. Histological studies of the tissues of mice with advanced leukaemia have revealed two types of infiltration with the malignant cells: lymphatic organs, such as lymph nodes or thymus, are rarely greatly enlarged, but show a massive replacement of their normal complement of lymphocytes by the malignant cells, their stromal architecture being preserved. Epithelial tissues, such as the liver, kidney and adrenal gland, show a massive occupation of their sinusoids or capillaries by the malignant cells; in general, neither the architecture of the organ nor the viability of the epithelial cells appears to be affected. Packing of the liver sinusoids is focal at an early stage but becomes generalised later (Fig. 1); a characteristic appearance is the concentration of malignant cell masses round the intermediate sized veins. Infiltration of the kidney decreases from the cortex to the medulla and is almost absent from the zone composed purely of collecting tubules. The adrenal gland becomes infiltrated from the periphery, the malignant cells passing inwards between the cortical columns of cells and spreading tangentially at the cortico-medullary junction; the medulla is free from malignant cells.

The histological appearance of the lungs in mice with advanced leukaemia is difficult to interpret. The alveolar architecture is well preserved but the alveolar walls show considerably increased cellularity and the presence of moderate numbers of mitotic figures. If these appearances are due to infiltration, it must be supposed that the malignant cells are contributing to the integrity of the alveolar walls. Although the presence of mitotic figures suggests the presence of malignant cells, the cytological features of the non-dividing cells in this situation do not permit of their reliable identification.

Histological examination of the brains of mice showing signs of nervous disturbance have revealed that, whilst malignant cells are to be seen round the vessels adjacent to the membranes, there is no infiltration of nervous tissue. The cerebellum has in all such cases examined shown multiple areas of degeneration involving the disappearance of many Purkinje cells; no inflammatory cells are seen, and it is assumed that the degenerative areas are infarcts associated with malignant cell emboli; one such embolised vessel has been seen adjacent to the pia mater, but such lesions are by no means frequent. These changes in the cerebellum, both by their situation and their extensiveness, are considered to be sufficient to account for the neurological signs in leukaemic mice. A zone of cerebellar degeneration is shown in Fig. 2.

The changes in the circulating leucocytes were studied in a group of CBA mice that had been injected intraperitoneally with $10^5$ leukaemia cells. At intervals after injection, total and differential leucocyte counts were performed on pairs of mice. Fig. 3 shows, for each cell type differentiated, the changes in the absolute cell count with time after injection. Each point represents the mean count for two mice. The absolute count has been expressed as the square root merely to facilitate

Fig. 3.—Changes in the circulating leucocytes at intervals after the intraperitoneal injection of malignant cells.

proportioning of the figure. Within 2 days after injection there is a sharp fall in the lymphocyte count, which returns to a normal level only after several days. This feature is a non-specific effect of intraperitoneal injection and has been regularly observed in control mice injected with suspensions prepared from normal liver and in other controls injected with the serum-Tyrode medium alone. Before the 14th day after injection no other significant change occurs. Between the 14th and 16th days there is a sharp rise in the lymphocytes, polymorphonuclear cells and monocytes ; by the 16th day, the lymphocytes and polymorphonuclear cells had attained 31,000 and 35,000 per c.mm. respectively. About one quarter of the cells recorded as polymorphonuclears on the 16th day were ring cells, often referred to as mouse myelocytes. Countable numbers of malignant cells were not found in the circulation until the 14th day, after which they rose rapidly to a level of about 12,000/c. mm. on the 16th day. It is recorded elsewhere that small numbers of malignant cells, detected by more sensitive transplantation methods, were found in the circulation by the 10th day after intraperitoneal injection of a similar mean cell dose. By the 16th day, the haemoglobin level of the injected mice had fallen to only 90 per cent of the level in uninjected control mice. The significance of the panleucocytosis observed in the leukaemic mice will be discussed later.

No systematic study of the bone marrow changes in this leukaemia has been made. Random examinations of the femoral marrow of leukaemic mice have not shown any consistent change: in one case marrow was replaced by malignant cells; in others, normal haematopoesis was found in the presence of occasional malignant cells; in one case an almost aplastic marrow was found. Evidently the marrow effects are irregular and possibly focal. The panleucocytosis and presence of immature forms of the granular cells suggests that extensive marrow replacement is not characteristic of the mice bearing the transplanted leukaemia.

*Characterisation of the malignant cells*

The appearance of the malignant cells by phase contrast microscopy has already been described under the description of the method of cell counting. Attempts by Professor R. J. V. Pulvertaft to cultivate the cells *in vitro* are still in progress. It has been found that the malignant cells do not survive *in vitro* for more than a few hours if cultivated alone; in the presence of moderate numbers of macrophages or of mouse embryo tissue their survival has been prolonged for several days, but no " permanent " cultures have yet been obtained. A similar dependence of cells on other cells, in tissue culture, has been described by de Bruyn, Korteweg and Waveren (1949).

In stained smears, prepared either from impressions of leukaemic organs or from the blood of leukaemic mice, the malignant cells are fairly large mononuclear cells with a deeply basophilic nucleus and cytoplasm; they bear a close resemblance to lymphoblasts. Their difference in size from lymphocytes, measurable though not very obvious in fresh suspensions, is quite obvious in the smears; this is to be expected if, in the process of making the smears, large and small cells are flattened to a uniform thickness. Small degenerate cells with intensely pycnotic and sometimes fragmented nuclei are regularly seen within groups of healthy-looking malignant cells, and are clearly identical with similar cells seen in histological sections of leukaemic tissues. Their random distribution among malignant cells suggests that degeneration is the result of intrinsic breakdown rather than of inimical tissue environmental factors.

A useful measure of the degree of replacement of normal cells by malignant cells in lymphatic tissue, or of the accumulation of malignant cells in other organs, can be got from study of the single-cell populations released from minced tissues. Single-cell suspensions were prepared from leukaemic organs and from homologous normal organs by the method already described. The diameters of 100 consecutive healthy-looking mononuclear cells were measured for each suspension, under phase contrast microscopy using a calibrated micrometer ocular, and frequency distribution curves of the diameters were constructed from the data. Some of these curves are reproduced in Fig. 4. Although the measurements were relatively crude by the method used, cells being measured only to the nearest 1·25 $\mu$, the results recorded illustrate clearly the differences between the single-cell populations obtained from homologous normal and leukaemic organs. The curves for the various leukaemic organs, when superimposed, are seen to be practically identical. The leukaemic thymus gland used was barely enlarged, and it will be appreciated that the diagram suggests a gross replacement of the normal by the malignant cells. The suspension prepared from normal liver contained only about 20 nucleated cells/c. mm.; that from leukaemic liver contained 5000/c. mm., and the frequency distribution curve suggested a very uniform population of cells judged by their size distribution. For this reason, leukaemic liver was usually used for the preparation of single-cell suspensions of the leukaemia cells. A similar uniform population of cells was obtained from the leukaemic kidney. This method of examining the cell population and of estimating the degree of replacement of normal by malignant cells in an organ has certain advantages over histological methods.

FIG. 4.—Frequency distribution of diameters of viable nucleated cells released from minced organs of normal and leukaemic mice.

●———● leukaemic organ.

○– – – –○ normal organ.

*Dissemination of the malignant cells from the site of injection*

For the purpose of interpreting the results of experimental therapeutic procedures, it was of some importance to know how soon after intraperitoneal injection the injected malignant cells were disseminated to the organs. The time of dissemination was studied by injecting groups of mice with leukaemic cells intraperitoneally and, after various intervals, injecting minced organs of these mice intraperitoneally into other CBA mice ; mice injected with the organ minces were observed for the development of leukaemia. All the injected mice were observed for a maximum period of 90 days, although leukaemic deaths rarely occurred more than 30 days after injection. All deaths recorded as leukaemic were confirmed as such by gross examination. Quantitative transplantation studies, to be described later in this paper, have shown that only very small numbers of cells are required to convey leukaemia to a mouse, so that this method of detecting dissemination is very sensitive.

Owing to limitation of the rate of supply of mice for experiment, these studies could not be made in a single experiment; therefore the experiments will be described separately.

*Changes in the free cell content of the peritoneal cavity.*—At intervals after the intraperitoneal injection of $10^5$–$10^6$ malignant cells into a group of mice, pairs of the injected mice were treated as follows: 2 ml. of serum-Tyrode was injected into the living mouse, and the abdomen was massaged for two minutes; the mouse was sacrificed and the peritoneal cavity opened; all free fluid was removed; the cavity was washed out twice with 1·0 ml. volumes of medium, and all the

FIG. 5.—Changes in the total nucleated cell count in washings of the peritoneal cavity, and in the survival times of mice receiving ⅓ of the cells recovered, at intervals after the intraperitoneal injection of 850,000 malignant cells into mice.

washings were pooled; the washings were centrifuged sufficiently to deposit all the cells, and the supernatant fluid was discarded; the cells were resuspended in 2·0 ml. of medium and well mixed, after which the density of total nucleated cells was determined by counting; 0·7 ml. of the suspension was then injected intraperitoneally into each of 2 CBA mice which were observed for the development of leukaemia. Attempts to do differential counts of the cells recovered from the peritoneal cavity were abandoned because of difficulty in obtaining reliable stained smear preparations. Table I shows, for each mouse investigated, the total number of cells harvested and the latent period between injection and death of the 2 CBA mice injected with the cells. The changes observed are illustrated graphically in Fig. 5. There is no significant change in the total count of the peritoneal cells between the 1st and 5th days, and it will be noted that, thereafter, the rise is not progressive. The changes in the mean survival time of mice injected with the cells suggest that there is a progressive rise in the number of malignant cells in the washings until at least the 10th day. These findings are not such as would be expected if a true ascitic tumour were being formed, and it may be noted here that none of several hundred mice injected intraperitoneally with about 5 million malignant cells has been found to have free fluid in the peritoneal space at the time of sacrifice or death. The fact that malignant cells are in the peritoneal cavity at all times after injection and therefore contaminate the surfaces of excised abdominal viscera, places great difficulty in the way of demonstrating the presence of malignant cells within these organs.

TABLE I.—*Cell Content of Peritoneal Cavities of Mice at Intervals After the Intraperitoneal Injection of* 850,000 *Cells*

| Time after injection | Total cells in washings (millions) | Survival times of mice injected with ⅓ of washings (days) |
|---|---|---|
| 2 hours | 20·0 | 17, 22 |
| | 20·8 | 19, 24 |
| 1 day | 3·1 | 24, 24 |
| | 8·0 | 23, 25 |
| 3 days | 4·0 | 22, 22 |
| | 4·0 | 21, 24 |
| 5 „ | 5·5 | 17, 22 |
| | 6·0 | 21, 21 |
| 10 „ | 57·5 | 14, 15 |
| | 32·0 | 13, 19 |
| 12 „ | 67·0 | 14, 15 |
| | 70·0 | 17, 20 |
| 14 „ | 52·0 | 16, 16 |
| | 55·0 | 16, 16 |
| 17 „ | 9·0 | 17, 18 |
| | 30·0 | 15, 15 |

*Dissemination to the blood.*—It has already been stated that countable numbers of malignant cells did not appear in the circulation until the 14th day after the intraperitoneal injection of $10^5$ cells into mice. In the present experiment the time of appearance of the malignant cells in the blood was investigated by the more sensitive method of transplantation. Of a group of mice that had been injected intraperitoneally with $10^5$ malignant cells, individual mice were treated at intervals after injection as follows : the terminal ¼ in. of the tail of the etherised mouse was amputated and the first drop of blood was discarded ; the next drop was used to determine the total leucocyte count ; the third and fourth drops were allowed to fall into a tube containing 0·5 ml. of serum-Tyrode containing 2·5 units/ml. of heparin, and the contents were mixed ; the weight of blood added was determined by weighing the tube and contents before and after addition of the blood. The volume of the mixture which contained 20 c.mm. of blood was calculated, and this volume of the diluted blood was injected intraperitoneally into each of one or more CBA mice, which were subsequently observed for the development of leukaemia. From the data recorded in Table II it will be seen that the 20 c. mm. samples of blood contained no demonstrable malignant cells until 10 days after injection of the donor mice. It will be noted also that although malignant cells were always present with a high leucocytosis, they were frequently demonstrable in the absence of leucocytosis. It is clear that the demonstration of malignant

TABLE II.—*Time of Appearance of Viable Malignant Cells in the Peripheral Blood of Mice After the Intraperitoneal Injection of* $10^5$ *of the Cells*

| Days after injection | T.W.B.C. of donor mouse | Fate of mice receiving 20 c. mm. of donor's blood* |
|---|---|---|
| 1 | 3,450 | S, S |
| 2 | 4,050 | S, S, S |
| | 3,925 | S, S, S |
| 3 | 4,450 | S, S, S |
| | 6,025 | S, S, S |
| 5 | 4,675 | S, S, S |
| | 7,500 | S, S, S |
| 6 | 7,500 | S, S, S |
| | 12,700 | S, S, S |
| 7 | 8,000 | S, S, S |
| | 7,800 | S, S, S |
| 8 | 11,400 | S, S, S |
| | 11,300 | S, S, S |
| 9 | 8,700 | S, S, S |
| | 11,100 | S, S, S |
| 10 | 10,100 | S, S, S |
| | 13,400 | 12, 23, S |
| 12 | 10,800 | 24, S |
| | 10,700 | S, S, S |
| 13 | 11,100 | 29, S, S |
| | 7,000 | 19, 24, 25 |
| 14 | 73,600 | 18, 18, 19 |
| | 9,250 | 24, S, S |
| 16 | 81 600 | 17, 17 |
| | 63,200 | 17, 19, 21 |
| | 8,500 | 18, 22, 24 |

* S = survival ; numbers indicate survival times of mice dying of leukaemia.

cells in organs at a time after the cells are known to enter the blood may indicate their presence in the contained blood rather than in extravascular sites.

*Dissemination to the lungs.*—Individual mice were sacrificed at intervals after their intraperitoneal injection with 150,000 cells. One lung was fixed for histological examination and the other was finely minced in a small volume of medium and injected into one or more CBA mice, which were subsequently observed for the development of leukaemia. The results of this experiment are recorded in Table III, from which it will be seen that malignant cells were not demonstrated in the lung until 9 days after injection. Significant infiltration with malignant cells was not detectable histologically in the fixed lungs until the 17th day. The results of the transplantation experiments with blood suggest that positive transplantation with lung tissue can sometimes be ascribed to the presence of malignant cells in the contained blood.

*Dissemination to spleen and liver.*—At intervals after the intraperitoneal injection of $10^5$ leukaemia cells into a group of mice, pairs of mice were sacrificed and treated as follows : the spleen, and a piece of the right lobe of the liver about the same size as the spleen, were excised and washed ; the liver fragments and spleens were pooled separately and minced finely in a small volume of medium. Each tissue pool was drawn into a syringe and used as two equal intraperitoneal inocula for two CBA mice, which were observed for the development of leukaemia. The tissue pools were derived from donor mice 3, 6, 8, 10 and 12 days after their injection. Malignant cells were thus demonstrated in all the spleen and liver

TABLE III.—*Detection of Malignant Cells in Lungs of Mice After their Intraperitoneal Injection with Malignant Cells*

| Days after injection | Fate of mice injected with lung tissue* | Presence of gross histological infiltration of contralateral lung with malignant cells |
|---|---|---|
| 1 | S | — |
| | S | — |
| 2 | S S | — |
| 4 | S S | — |
| | S | — |
| 5 | S, S | — |
| 7 | S, S | — |
| | S, S | — |
| 8 | S, S | — |
| | S, S | — |
| 9 | 21, 21 | — |
| | 27 | — |
| 10 | S, S | — |
| | 33, 35 | — |
| 11 | 26, S | — |
| | 21, 22 | — |
| 12 | 23, 25 | — |
| 14 | 23, 27 | — |
| | 22, 22 | (Bronchial gland only) |
| 15 | 22, 22 | — |
| 16 | 16, 16 | — |
| 17 | 17, 17 | + |
| 18 | 15, 16 | + |

* S = mouse surviving : numbers indicate survival times of mice dying with leukaemia.

suspensions except the liver preparation from mice 3 days after injection. These results were predictable from the findings for the peritoneal washings, and it must be assumed that the malignant cells demonstrated in these organs were often those contaminating the peritoneal surface.

*Dissemination to the axillary lymph nodes.*—At various intervals after the intraperitoneal injection of a group of mice with $10^5$ leukaemia cells, individual injected mice were sacrificed and treated as follows : both axillary regions were exposed by incisions which did not extend below the costal margins, and the most obvious presenting axillary node on each side was excised ; the glands were finely minced in about 0·2 ml. of medium and injected intraperitoneally into a CBA mouse which was observed for the development of leukaemia ; two donor mice were treated thus on every day from the 1st to the 12th day after their injection. Transplantable leukaemia cells were demonstrated in the axillary glands of all mice from the 7th day onward, except in those from one mouse sacrificed on the 9th day.

A comprehensive picture of the time of dissemination to the various tissues and organs is given in Table IV. It is evident that a generalised dissemination of the malignant cells from the peritoneal cavity was not demonstrated until about one week after the intraperitoneal injection of mice. The fairly long period before detectable numbers of malignant cells appear in the circulation suggests that their appearance may only follow reproduction of the cells in an extravascular situation with the production of lesions which provide access for the cells to the circulation. These aspects of dissemination are to be discussed later.

TABLE IV.—*Demonstration, by Transplantation Methods, of Malignant Cells in Various Sites at Intervals After the Intraperitoneal Injection of* $10^5$–$10^6$ *of the Cells*

| Site | Days after injection | | | | | | | | | | | | | | | | |
|---|---|---|---|---|---|---|---|---|---|---|---|---|---|---|---|---|---|
| | 1 | 2 | 3 | 4 | 5 | 6 | 7 | 8 | 9 | 10 | 11 | 12 | 13 | 14 | 15 | 16 | 17 |
| Blood (20 c. mm.) | − | − | − | .. | − | − | − | − | − | + | .. | ± | + | + | .. | .. | .. |
| Peritoneal cavity | + | .. | + | .. | + | .. | .. | .. | .. | + | .. | + | .. | + | .. | .. | .. |
| Spleen (whole) | .. | .. | + | .. | .. | + | .. | + | .. | + | .. | + | .. | .. | .. | .. | .. |
| Liver (0·3–0·4 g.) | .. | .. | − | .. | .. | + | .. | + | .. | + | .. | + | .. | .. | .. | .. | .. |
| Lung (whole) | − | − | .. | − | − | .. | − | − | + | ± | + | + | .. | + | + | + | + |
| Axillary glands (2) | − | − | − | − | − | − | + | + | ± | + | + | + | .. | .. | .. | .. | .. |

± Indicates conflicting results using two equivalent donor mice.

*Attempts to modify the survival time of mice after intraperitoneal injection with leukaemia cells*

It was of interest to know whether the survival times of injected mice could be modified significantly by surgical or other interference, and if so, whether this was associated with an alteration of the rate of dissemination of the cells.

*Effect of preliminary splenectomy.*—Seventy-two male and female CBA mice were divided into two equivalent groups. Mice of one group were splenectomised under ether anaesthesia ; a fake operation, involving delivery and return of the spleen, was performed on mice of the other group. Operations were performed alternately. Three days after operation, all the mice were injected intraperitoneally with $10^5$ malignant cells, and the times of death of the injected mice were recorded. All deaths were confirmed as leukaemic. The mean time of death of the splenectomised mice was 19·4 days ; that of the fake-operated controls was 20·6 days. The difference is not significant.

*Effect of treatment with massive doses of radiation-killed leukaemia cells.*—A dense, washed suspension of the malignant cells, prepared from numerous livers and spleens of mice with advanced leukaemia, was exposed to 14,000 r of 2-MeV radiation. A control suspension from normal livers and spleens was similarly prepared and irradiated. Twenty female CBA mice were each injected intraperitoneally with $10^5$ viable leukaemia cells. Ten of the injected mice received daily intraperitoneal injections of the irradiated malignant cell suspension from the 4th to the 7th day after receiving the viable malignant cells. The other ten mice received similar volumes of the control suspension at the same times. Differential white blood counts done on the 12th and 14th days after the injection of viable cells showed no differences between the two groups of mice, and all mice of both groups examined showed circulating malignant cells on the 14th day. The mean survival times of the two groups were not significantly different, being 19·5 days and 20·4 days. The total number of radiation-killed malignant cells received by the mice was $5 \times 10^8$, and it is of considerable interest that this overwhelming preponderance of dead cells had no detectable influence on reproduction of, or invasion by, the viable cells.

*The effect of cortisone administered during the course of the transplanted disease.*—Two groups of 10 mice were injected intraperitoneally with $10^5$ malignant cells. From the 7th day after injection until death each mouse of one group received daily subcutaneous injections of 1 mg. of cortisone acetate in 0·04 ml. of vehicle.

Mice of the control group received daily injections of 0·04 ml. of saline over the same period.

The results of differential blood counts performed on all mice of the two groups on the 12th day after injection of the malignant cells are given in Table V. In addition to the expected lymphopoenia and eosinopoenia of the cortisone-treated mice there is an indication that dissemination of the malignant cells to the circulation had been delayed by the treatment. The mean survival time of the treated group was 19·4 days, compared with 16·2 days in the control group. Although there is a suggestion that the disease was restrained in the treated mice, the difference in the mean survival times is not significant.

TABLE V.—*Effect of Cortisone Treatment on Differential Leucocyte Counts of Mice After their Intraperitoneal Injection with Malignant Cells*

| | Mean % differential count (range) | | | | |
|---|---|---|---|---|---|
| | Lymphocytes | Polymorphs | Monocytes | Eosinophils | Malignant cells |
| Controls . . | 31·8 (23–41) | 49·5 (40–62) | 14·2 (7–21) | 3·7 (1–7) | 0·8 (0–4) |
| Cortisone-treated . | 7·6 (4–15) | 91·8 (85–96) | 0·6 (0–2) | 0 | 0 |

*Quantitative transplantation of the leukaemia*

The experiments to be described in this section were undertaken for the following purposes : to assess the sensitivity of transplantation as a method for detecting the presence of the malignant cells in an inoculum, as was done in the studies of dissemination ; to indicate the proportion of morphologically intact leukaemia cells which are capable of initiating the disease on transplantation ; and to provide the basis for radiobiological experiments in which the proportion of leukaemia cells inactivated by various doses of radiation was to be determined.

*Comparison of TD50 values obtained for different sites of injection.*—Using 4 groups of 5 mice, and injecting the groups with mean malignant cell doses falling in tenfold dilutions from 500 to 0·5, the following TD50 values were obtained for titrations done by injecting cells into the sites indicated :

Intravenously, 1·6 cells
Intraperitoneally, 1·2 cells
Subcutaneously, 3·5 cells

These differences are not significant, and it is concluded, in view of the very different environments in which the injected cells were deposited, that the transplantation hazard to the cells is very slight. Since the intraperitoneal route is technically the simplest and is not inferior to the intravenous route in sensitivity, it has been adopted as the route of choice for titration purposes.

*Effect of sex of hosts on the TD50 values obtained in intraperitoneal titrations.*—This study was undertaken merely to confirm that male or female mice could be used indiscriminately for titration purposes.

A leukaemia cell suspension was titrated intraperitoneally in male and female mice of the same age. For each sex, there were 4 groups of 12 mice ; the mean numbers of cells injected into the groups fell in tenfold steps from 2000 to 2 cells ; mice of the two sexes were injected alternately.

The TD50 values obtained for the two sexes were : males 8·0 cells ; females, 5·6 cells. The difference is not significant.

*The effect on the viability of malignant cells of storage for short periods at 0–2° C.*—It will be appreciated that the time between preparation of a leukaemia cell suspension and injection of dilutions into mice varies with the particular intervening procedures involved. Although, in all experiments, the cells were kept at 0–2° C. throughout this period, it was of importance to know whether any measurable loss of viability of the cells occurred in the longest period likely to elapse between preparation and injection of a suspension.

Serial tenfold dilutions of a leukaemia cell suspension prepared in the usual way were made in 5 per cent serum-Tyrode, to give suspensions having 2000, 200, 20 and 2 mean cells/0·1 ml. Each of these dilute suspensions was distributed into 3 tubes to give three similar series of dilutions for titration. The three series of dilutions were injected into mice, 2, 5 or 8 hours after killing the donor mouse, the last two series being kept at 0–2° C. until injected. For each titration there were 4 groups of mice, each group consisting of 4 males and 4 females. All injections were intraperitoneal, and the volume injected was always 0·1 ml.

The TD50 values obtained for the three titrations were as follows :—

After 2 hours, 8 cells
After 5 hours, 4 cells
After 8 hours, 9 cells

These differences are not significant, and it is concluded that the viability of the malignant cells is not measurably impaired by storage at 0–2° C. for at least 6 hours.

The undiluted single-cell suspension used in the above experiment was resuspended and recounted after a total period of 23 hours' storage at 0–2° C. The density of morphologically intact malignant cells was not significantly reduced. In related experiments, the density of morphologically intact cells in a suspension was determined at intervals after longer periods of storage at 2° C. It was found that a reduction to one-tenth of the starting density was not attained until after about 5 days. The deterioration rate was not significantly influenced by the medium in which the cells were suspended—whether mouse serum, 10 per cent serum-Tyrode solution, or physiological saline. From these findings, it appears that the malignant cells may be capable of resisting a relatively inimical environment for fairly long periods at temperatures just above freezing.

*Repeatability of intraperitoneal titrations.*—The investigations of the influence of sex and of storage of the cells, which are described above, were done in a single experiment, and it will be noticed that the TD50 values obtained were in the range 4–9 cells. In this experiment, however, an unusual amount of pipetting of the suspensions was required in order to keep the cells in suspension in the course of the experiment. This treatment would be expected to reduce the viability of the cells by trauma, and to be responsible for giving TD50 values higher than would be obtained with gentler treatment of the cells.

Table VI shows the conditions and results for 6 intraperitoneal titrations, performed under standard conditions, of malignant cell suspensions prepared from leukaemic livers. It will be seen that the TD50 values obtained were all within the range 0·7–3 cells ; the mean of the 6 values was 2·0 cells. It will be noted that a titration performed in mice that were mostly over one year old gave a typical result, indicating that no restriction of the power of the malignant cells to reproduce or invade occurs in older mice.

TABLE VI.—*Details of Six Intraperitoneal Titrations of Malignant Cells derived from Infiltrated Livers*

| Serial passage number | Sex of mice | Age of mice (days) | Mice per group | Mean cells injected (range) | Result TD50 |
|---|---|---|---|---|---|
| 42 | ♀ | 96–183 | 5 | 0·5–500 | 1·2 |
| 49 | ♀ | 98–128 | 5 | 1–1000 | 3·0 |
| 54 | ♂ | 145–172 | 6 | 1–1000 | 0·7 |
| 58 | ♀ | 141–158 | 6 | 0·08–10 | 2·0 |
| 63 | ♂ | 184–195 | 10 | 0·2–25 | 3·0 |
| 70 | ♂ | 358–424 | 10 | 0·17–27 | 2·1 |

Mean TD50 : 2·0 cells.

Titration methods involving quantal responses in small groups of animals are of intrinsically low precision (Meynell, 1957). The small variation in the TD50 values obtained for the 6 titrations is therefore well within the experimental error.

FIG. 6.—Relationship between log mean malignant cell dose injected intraperitoneally, and incidence of leukaemia in the injected mice. Data of 6 titrations.

In Fig. 6, the data from the 6 titrations have been used to construct a graph of the relationship between mean cell dose injected (expressed as the logarithm) and the percentage of injected mice developing leukaemia. It will be seen that there is no significant departure from the theoretical Poisson curve drawn through the mean TD50 for the 6 titrations. The TD63, both from the curve and from the mean of the TD63 values obtained in the 6 titrations, is 3·0 cells.

*Titration of circulating malignant cells.*—Stained blood smears from mice with advanced leukaemia showed three types of abnormal leucocyte. These were: intact large mononuclear cells with unlobed and non-indented nuclei of fine texture ; large " smudge " cells, which were taken to be cells of the first type that had been damaged in the process of making the smear ; and circular or oval cells only slightly larger than erythrocytes and having intensely pycnotic, and sometimes fragmented, nuclei. Cells of the first variety were designated viable malignant cells ; the existence of the second type indicated that differential counts made from stained smears would tend to underestimate the percentage contributed by intact leukaemia cells to the total leucocyte count ; the small pycnotic cells resembled those seen within groups of leukaemia cells in sections of leukaemic tissues and are almost certainly degenerate leukaemia cells.

To avoid the difficulties associated with cell damage during the making of blood smears, the density of morphologically intact leukaemia cells in the circulating blood was determined by counting the cells directly by phase-contrast microscopy in blood diluted in leucocyte counting fluid consisting of N/10 HCl. Under these conditions the leukaemia cells are seen to have perfectly spherical nuclei with a regular chromatin pattern. An impression of their distinctive character was soon formed by repeated comparison with the appearances of normal leucocytes.

The titratable activity of the circulating leukaemia cells was measured in order to check their morphological recognition and to provide a comparison with the liver leukaemia cells. It was of particular importance to know whether the leukaemia cells derived from minced leukaemic liver were different, with respect to their ability to give rise to leukaemia after transplantation, from leukaemia cells situated in other sites.

Under ether anaesthesia, the tail of a leukaemic mouse was amputated half an inch from the tip. The first drop of blood was discarded and the following two drops were allowed to fall into 2 ml. of chilled serum-Tyrode medium containing 2 units/ml. of heparin. After thorough mixing, an aliquot of the diluted blood was further diluted 1/10 in leucocyte counting fluid and the density of leukaemia cells in the diluted blood was determined. Further, serial, dilutions of the blood were injected intraperitoneally into groups of mice. The TD50, calculated in the usual way, was 1·8 cells, which is close to the mean obtained for the 6 titrations of liver leukaemia cells reported above. It is concluded that the circulating malignant cells were correctly identified and that their viability was not significantly different from that of the liver leukaemia cells.

*Survival times of mice after injection with leukaemia cells*

Fig. 7 is a scatter diagram, constructed from the data of numerous intraperitoneal titrations, each point of which represents for an individual mouse the relationship between log mean cell dose injected and survival time. To standardise the results from different experiments, the mean cell dose has been recorded as a factor of the TD50 obtained in the titration in which a particular mouse was used. It is clear from the diagram that the latent period after the injection of a given dose of cells is very variable from mouse to mouse over the range of mean cell doses shown and cannot be used to estimate the cell dose.

Among 130 mice which received several million leukaemia cells intraperitoneally the survival time ranged from 9 to 20 days ; both the mean and modal survival times were 13 days.

A comparison of the data from intravenous, intraperitoneal and subcutaneous titrations showed, in that order, a progressive increase of variability of the survival time among mice given the same mean cell dose. In the case of the intravenously injected mice, the survival times of mice receiving the same mean cell dose did not vary by more than 2 days, and there was a regular reduction of about 24 hours in the mean survival time of injected mice for each tenfold increase of mean cell dose. If, as may reasonably be supposed, death of a mouse occurs when the total number of leukaemia cells present in the body has reached a certain critical level, we may conclude that in 24 hours there is a tenfold increase of the cells present.

FIG. 7.—Scatter diagram of relationship between log mean malignant cell dose injected intraperitoneally, and survival times of individual injected mice.

This corresponds to a generation time of about 8 hours. Assuming a period of 40 minutes for the duration of mitosis, a cell population undergoing increase at this rate should show about 8 per cent of the cells in mitosis. Mitotic rates approaching this figure have, in fact, been found for this leukaemia in histological sections of the infiltrated liver.

*Homologous transplantation of the leukaemia*

Only one foreign strain of mouse, a heterozygous albino strain, was available for attempted homotransplantation of the leukaemia. Twelve adults of this strain showed no sign of sickness after the intraperitoneal injection of large doses of the leukaemia cells. CBA × albino $F_1$ hybrids, on the other hand, regularly died with leukaemia after intraperitoneal injection with the cells, the course of the disease being indistinguishable from that in the CBA mice.

Each of a litter of 7 albino embryos was injected with CBA leukaemia cells *in utero* 3–4 days before birth. One death of unspecified cause occurred 6 days after injection. On the 38th day after injection, 2 of the mice were seen to have tumours in the region of the neck and ear. These mice were killed and examined. The neck masses were in both cases composed of cells indistinguishable from the leukaemia cells. In one case, there was, in addition, massive infiltration of the liver and spleen ; in the other, there was no gross enlargement of spleen or liver but a small mass of malignant cells was found infiltrating one ovary. The remaining 4 injected albino mice were found to be free from any gross evidence of disease when killed 120 days after injection. Local masses of the malignant cells, such as were seen in some of the albino mice, have never been seen in leukaemia-bearing CBA mice, even after subcutaneous injection.

*Attempted cell-free transfer of the leukaemia*

It is clear that the results of the quantitative transplantation studies would require careful interpretation if there were any question of cell-free transfer of the malignant condition. On the other hand, the fact that the end-points of the titrations were so regularly in the region of 2 cells made it most improbable that there was any transmission by subcellular elements. Of 7 adult female CBA mice injected intraperitoneally with centrifuged concentrated extract of leukaemic liver, 2 died of other causes 8 and 18 months respectively, after injection ; the remaining 5 are alive and well after 2 years.

Six CBA males were injected intraperitoneally with $10^8$ morphologically intact leukaemia cells that had been exposed to 14,000 r of 2-MeV radiation, a treatment which would not be expected to reduce significantly the activity of any virus-like agent. All 6 mice are alive and well 18 months after injection.

The very low incidence of spontaneous leukaemia in CBA mice made it most improbable that any pre-natal transmission of a leukaemia-inducing subcellular factor, as demonstrated in mice of the AK strain (Gross, 1954), is involved in the leukaemic condition described here. Nevertheless, the demonstration of such a factor in the CBA leukaemia cells would require the injection of cell-free extracts into embryos or mice near to term. Sixteen CBA embryos were injected *in utero* with concentrated and centrifuged, but not filtered, extract of the leukaemia cells. The injected mice were all born 24–48 hours after injection. All the mice survived one year, when most of them died or were killed as the result of a localised epidemic of liver infection with *B. piliformis* ; none showed evidence of leukaemia. Two are surviving 2 years after injection.

As previously stated, about one-quarter of the leukaemic mice developed gross signs of neurological disorder a few days before death, and this is associated with degenerative lesions in the cerebellum. To exclude the possibility that these features were due to the activity of a neurotropic passenger virus, the brains of affected mice have on several occasions been used to prepare cell-free extracts which were injected intracerebrally or intraperitoneally into other mice. Mice so treated have not shown any evidence of neurological disturbance and have not developed leukaemia.

Thus, numerous experiments have all failed to reveal a subcellular factor capable of transmitting either the leukaemia of its neurological manifestations. Moreover, the methods employed in these experiments for eliminating viable cells from the extracts used (centrifugation or irradiation) were such as would not be expected to reduce significantly the activity of any virus-like agent.

DISCUSSION

It will be recalled that the original mouse with spontaneous leukaemia exhibited gross enlargement of the lymph nodes and a marked degree of anaemia, features which have not been seen in any mouse with the transplanted disease. Two possible explanations for these differences present themselves. Firstly, the leukaemia of the original mouse may have started as a relatively benign neoplastic condition, during which the anaemia and nodal enlargement had time to occur; in the course of the disease in this mouse a progression of the malignancy may have occurred suddenly so that the cell population transplanted in the first passage was dominated by cells which had acquired enhanced malignancy. Alternatively, the leukaemia of the original mouse may have arisen as a late complication of some insidious non-transplantable disorder of the reticulo-endothelial system. Owing to the rarity of spontaneous leukaemia in the CBA strain no further studies of these interesting differences can be made. The matter is of some interest, however, in relation to possible aetiological factors leading to leukaemia in the first mouse.

The panleucocytosis occurring in the terminal stages of the transplanted disease is not easily explained. The rise in both granulocytes and monocytes argues against there being an extensive replacement of the haemopoetic tissue by malignant cells. Indeed, the appearance of fairly numerous myelocytes suggests that there is some stimulation of the marrow; and it is possible that this is brought about by breakdown products from the large numbers of malignant cells which undergo degeneration. Most difficult to explain is the high lymphocytosis. Since the lymphopoetic tissues studied were not only enlarged but also extensively replaced by malignant cells, a reduction of the input of lymphocytes into the circulation is to be expected. These findings are not be explained by errors of distinction between the lymphocytes and the malignant cells. In stained smears the distinction is clear; and the result of the titration of circulating malignant cells, being almost identical with that of the titrations of uniform populations of malignant cells from leukaemic livers, showed that a reliable distinction had been made. In considering possible explanations of the panleucocytosis, it is useful to recall that the peripheral leucocyte count may be the resultant of a dynamic process in which cells are constantly entering the circulation from their sites of production and being removed at sites of sequestration (Bierman, Kelly and Cordes, 1955). Since the panleucocytosis is not satisfactorily explainable in terms of increased leucocyte production, it is reasonable to suggest that blocking of sequestration sites by malignant invasion may have much to do with the terminal changes in the circulating leucocyte counts. Such interference with the sequestration sites is certainly to be expected if the mechanisms at these sites succeed in attracting the malignant cells but fail to dispose of them.

Among the 42 tumours which Klein and Klein (1956) attempted to convert to growth in the ascitic form, none was described as a leukaemia. There were, however, 5 lymphomas, presumably composed of a cell type similar to that of the tumour described here; all of these lymphomas formed ascitic tumours after their first intraperitoneal passage. In the case of the present tumour, there was an increase in the intraperitoneal population of leukaemia cells after intraperitoneal injection, but this was not progressive. None of several hundred mice injected intraperitoneally with large numbers of the malignant cells has shown free fluid in the cavity. Thus, although the cells were capable of multiplication in the cavity, they neither attracted free fluid to it nor were retained in it. Among the properties required for growth in the ascites form, rapid growth rate, reduced intercellular adhesion, and ability to withstand relatively anoxic conditions have been suggested. It might be added that a high propensity for dispersal from the site of injection would tend to discourage the formation of ascites tumours. The tendency of the cells of the present leukaemia to disperse from the site of injection is displayed also after subcutaneous or intramuscular injection ; all of numerous injections at these sites have failed to produce palpable masses. It is indeed remarkable that cells of comparable capacity to form free cell suspensions, such as those of sarcoma 37, show a strong tendency to proliferate into solid masses at subcutaneous sites of injection.

The dissemination experiments have not disclosed the route taken by the malignant cells after leaving the site of injection. After injection of a large inoculum of malignant cells into the peritoneal cavity, there appears to be no significant change in the cell content of the cavity for at least 5 days, and no malignant cells were demonstrated in organs outside the cavity for 7 days. Failure to demonstrate the cells in the lungs or blood till after 9 or 10 days seems to imply that detectable numbers of the cells are not entering the veins until a relatively late stage in the progress of the disease. Further studies of the route of dissemination are in progress, but the following tentative hypothesis is proposed to explain the behaviour of the cells on the basis of the limited experiments reported here and of the route of lymph drainage from the peritoneal cavity described by Yoffey and Courtice (1956) : it is proposed that numbers of the injected cells are constantly removed from the peritoneal cavity to the diaphragmatic lymph plexus ; that they pass thence, via the collecting channels running with the internal mammary vessels, to glands in the anterior mediastinum ; there, the cells are retained and undergo multiplication, eventually replacing the normal lymphatic tissue ; at a late stage of the pathological changes in these glands large numbers of malignant cells are discharged into the thoracic duct or right lymphatic duct and thence into the circulation. At this stage an opportunity occurs for invasion of various organs. On the other hand, it appears that some mechanism operates to remove malignant cells from the circulation : when leukaemic cells were injected intravenously in a quantity sufficient to give easily detectable numbers in blood smears if they had remained in circulation, only an occasional cell could be seen in blood smears taken at intervals of between a few seconds and one hour after intravenous injection. This finding, considered along with the fact that considerable numbers of the malignant cells persist in the circulation of mice at a late stage of the disease, suggests that the density of the circulating malignant cells in mice with advanced leukaemia may be the result of a continual input of cells from the lymphatic tissue and a simultaneous continual withdrawal of the cells in other situations.

The results of the present studies of dissemination have indicated that the distribution of the cells during the first week after intraperitoneal injection is a matter requiring further detailed study. It should be mentioned here that Skipper *et al.* (1957) demonstrated mouse leukaemia cells by transplantation methods in the spleen, liver, lymph nodes, lungs and blood of mice that had been injected intraperitoneally 24 hours previously with 20,000 or more cells of an AK leukaemia. The inoculum of tissue was in each case a 100 mg. aliquot of a pool from a group of similarly treated mice, so that comparison of these results with those presented here is difficult. However, these authors do not mention, even in the case of the spleen and liver, what steps were taken to prevent contamination of their inocula with malignant cells free in the peritoneal cavity, an omission which detracts from the value of the data they give as evidence of actual dissemination to the tissues they investigated.

The failure of injections of very large doses of radiation-killed leukaemia cells to influence the progress of the transplanted disease contrasts with the results both of Revesz (1955) and Scott (1957). Revesz, using the Ehrlich ascites tumour, found that the addition of radiation-killed cells to viable cell inocula stimulated the growth of ascites tumours from large inocula but inhibited the growth from small inocula. Scott, using the same tumour, found that the growth of ascites tumours from both small and large inocula of viable cells was stimulated by the presence of radiation-killed cells. Hewitt (1953) found slight evidence of an increase of the incidence of solid sarcoma 37 tumours from small viable cell inocula when these were mixed with a preponderance of sarcoma 37 cells killed by freezing. Since the experiments reported here with CBA mice were made using a genetically pure tumour-host system, and are in this respect distinctive, it is possible that the stimulation or inhibition effects observed in the experiments referred to were manifestations of immunity factors.

Failure to effect significant modification of the course of the transplanted disease by cortisone treatment of the mice is a finding which conforms to clinical experience with the cortisone treatment of acute lymphatic leukaemia in children (Mider, 1957).

Fig. 6, constructed from the data of 6 intraperitoneal titrations, shows the relationship between the mean number of morphologically intact leukaemia cells injected intraperitoneally and the proportion of injected mice subsequently dying of leukaemia. It will be seen that, within the limits of experimental error, the plotted points conform to the theoretical Poisson curve. When, as here, the conditions of sampling justify the use of the Poisson series, the chance of obtaining at random a sample (inoculum) containing no " taking unit " for successful transplantation of the leukaemia, is connected by a calculable relation with the mean number of such units per sample. This chance is given as $c^{-m}$, where $m$ is the mean number of units per sample (Fisher, 1950). When $m = 1$, $c^{-m} = 37$ per cent. From Fig. 6, a suspension which gives 37 per cent of failures (63 per cent of takes) contains a mean of about 3 morphologically intact malignant cells. This means that, of every 3 cells in an inoculum only 1 is likely to be able to give rise to a fresh leukaemia cell population. An important consideration here is the cause of the failure of two-thirds of the apparently viable leukaemia cells to convey the leukaemia when transplanted. It is possible that this proportion represents cells which were damaged by the pipetting necessary for mixing and making the dilutions. Cells in the final dilutions of a series will have been sucked up and discharged from a pipette about fifty times, and it seems likely that such treatment would reduce the viable cell count ; moreover, high TD50 values were obtained in titrations in which the pipetting was greater than usual. A second possibility is that a proportion of the injected cells were deposited in an inimical situation. In view of the fact that no significant differences were observed between the TD50 values obtained for titration of a suspension by the intravenous, intraperitoneal and subcutaneous routes, this explanation seems improbable. Since transplanted tissue will proliferate when confined in micropore filters (Algire, Weaver and Prehn, 1954) it seems most unlikely that injected single cells could find themselves in any part of the tissues where they would fail to make contact with their requirements. Evidence has been presented elsewhere (Hewitt, 1956) which suggests that the hazard to healthy isolated malignant cells, once they have been injected, is probably insignificant. Apart from these possible hazards to the cells involved in their handling during the transplantation procedure, there remains the possibility that a proportion of the cells which pass as viable cells in the counts are, in fact, intrinsically defective, having arisen from a disordered mitosis and being incapable of giving rise to a clone in any circumstances. That such " doomed " cells do arise in the leukaemia cell population there is little doubt. Histological examination of leukaemic tissues reveals that, apart from zones of apparently dead cells (which may be small areas of infarction), there is, among the healthy malignant cells, a random distribution of cells displaying pycnosis or karyorrhexis. These form a fairly constant proportion of the malignant cells and are to be seen in all infiltrated tissues and in the blood of leukaemic mice. Pycnotic cells are not infrequently seen adjacent to mitosing cells, and it is concluded that degeneration of the cells is determined by intrinsic defects. Since the length of time for which such cells remain in the tissues is not known, the frequency with which they arise cannot be stated. It is clear, however, that a comprehension of the population changes in the malignant cells requires a consideration of the frequency of such intrinsically defective cells. It may be noted that the TD50 values reported here for the CBA leukaemia are considerably less than have been obtained by other workers in similar experiments with other strains of mouse leukaemia. Furth and Kahn (1937), in their classical study of the quantitative transplantation of mouse leukaemia, obtained only 5 per cent of takes in 97 transplantations of single leukaemic cells, whereas in the present experiments 25 per cent of takes were obtained for inocula calculated to contain an average of one cell. Goldin *et al.* (1954) give a dose response curve for transplantation of a mouse leukaemia which shows a TD50 value of about 1000 cells, compared with 2 cells obtained here for the CBA leukaemia. Comparisons of this kind can be made usefully only if identical techniques are used in the two experiments. Technical differences relating to the medium used for dilution purposes, the temperature at which the dilutions are carried out, the time elapsing between killing the donor mouse and injecting the cells, and the method of preparation of the single-cell suspensions, would be expected to have a considerable influence on the viability of the explanted cells and thus on the results of viable cell titrations. The following technical details of the titration procedure used in this laboratory are aimed at reducing to a minimum possible unfavourable influences on the cells : the use of hand mincing by scissors to release the single cells, this having been found to be far less damaging than methods involving incisive cuts by sharp blades ; a fully aseptic technique ; the use of a protein-containing medium for the dilutions ; reduction to a minimum of the time between preparation of the cell suspension and injection of cells ; the use of pipettes having a relatively wide bore even at the tip, for mixing and diluting ; and keeping the cells cold throughout the *in vitro* procedures.

Sixteen mice which were injected *in utero* with centrifuged extracts of CBA leukaemia cells survived for one year without developing leukaemia. By contrast, Gross (1954) records that, of 41 C3H or C3Hf mice which were injected with centrifuged extracts of AK leukaemic tissue, almost half developed leukaemia at an average age of 5½ months. Thus, there is no analogy, in this respect, between the AK leukaemia and the CBA leukaemia described here. It is clear that non-cellular transmission of a leukaemia is less likely to be encountered in circumstances where no " vertical " transmission of the leukaemia from generation to generation of an inbred strain has been observed. Until non-cellular transmission has been demonstrated for leukaemias arising in mice of strains other than the AK strain, as was attempted here, no general statement about the aetiology of mouse leukaemia appears to be permissible. Investigating the aetiology of a disease in which an extrinsic agent is known to play a decisive role is, both technically and intellectually, a more promising undertaking than the investigation of conditions in which no such agent is likely to be demonstrable. It is for this reason, perhaps, that there is a tendency to extend the concept of an extrinsic agent far beyond the limited circumstances in which such a factor has been found. As far as mouse leukaemia is concerned, it is useful to maintain the perspective of the subject by insisting upon the uniqueness of the AK leukaemia situation until such time as one or more extrinsic factors can be demonstrated in sporadic cases of leukaemia occurring in mice of other strains.

### SUMMARY

1. Differences are described between the pathological findings in a CBA mouse with spontaneous lymphocytic leukaemia and those in CBA mice to which the leukaemia was subsequently transplanted. The significance of these differences is discussed in relation to genesis of the leukaemia in the original mouse.

2. The typical course of the transplanted disease is described with particular reference to the changes in the circulating leucocytes.

3. Signs of severe disturbance of the central nervous system, occurring in one-quarter of the mice bearing the transplanted disease, were associated with foci of degeneration in the cerebellum ; no malignant infiltration of the brain was observed. No evidence of an associated neurotropic virus infection was obtained.

4. The malignant cells resembled the larger cells of the normal thymus gland. The diameters of the malignant cells gave a characteristic frequency distribution curve which was readily distinguishable from that of the nucleated cells released from normal organs such as the spleen, thymus or lymph gland. The construction of such curves provided a means of demonstrating the degree of replacement by malignant cells in lymphoid tissue.

5. After injection of malignant cells into the peritoneal cavity, there was an initial increase in the number of malignant cells in the cavity but this did not progress and no ascites tumours were produced. Solid tumours were not seen after the subcutaneous injection of malignant cells.

6. After the intraperitoneal injection of $10^5$ malignant cells, transplantable malignant cells were not demonstrated in the blood, lungs or axillary glands of the injected mice until at least 1 week after injection.

7. The mean survival time of mice injected with malignant cells was not modified by splenectomy three days *before*, or by administration of cortisone or the injection of large numbers of radiation-killed malignant cells *after*, the injection of viable malignant cells.

8. A method is described for the " titration " or bioassay of suspensions of viable malignant cells, the results of a titration being expressed as the TD50, that is the number of apparently viable malignant cells required to convey leukaemia to 50 per cent of a group of injected mice. The TD50 values obtained in 6 separate intraperitoneal titrations of cells derived from leukaemic livers varied from 0·7 to 3·0 cells (mean, 2·0 cells). The following factors had no significant influence on the TD50 values : the route of injection of the cells (intravenous, intraperitoneal or subcutaneous) ; storage of the cells for 8 hours at 0–2° C. before injection ; or the source of the leukaemia cells (leukaemic liver or blood).

9. The leukaemia was not transplantable to adult mice of a heterozygous albino strain but was successfully transplanted to a proportion of the albinos by their injection *in utero*.

10. The results of several experiments, including the injection of centrifuged extracts of leukaemic tissues into CBA mice *in utero*, provided no evidence of cell-free transmission of the leukaemia.

I am indebted to Mrs. Dorothy Levy for her skilled technical assistance, and to Dr. P. Hansell and the Department of Medical Photography, Westminster Hospital for the photomicrographs. I am grateful for grants from the British Empire Cancer Campaign, during tenure of which this work was done.

### REFERENCES

ALGIRE, G. H., WEAVER, J. M. AND PREHN, R. T.—(1954) *J. nat. Cancer Inst.*, **15**, 493.
BIERMAN, H. R., KELLY, K. H. AND CORDES, F. L.—(1955) *Ann. N.Y. Acad. Sci.*, **59**, 850.
DE BRUYN, W. M., KORTEWEG, R. AND VAN WAVEREN, E. K.—(1949) *Cancer Res.*, **9**, 282.
FISHER, R. A.—(1950) 'Statistical Methods for Research Workers'. Edinburgh (Oliver and Boyd), p. 61.
FURTH, J. AND KAHN, M. C.—(1937) *Amer. J. Cancer*, **31**, 276.
GOLDIN, A., VENDITTI, J. M., HUMPHREYS, S. R., DENNIS, D., MANTEL, N. AND GREENHOUSE, S. W.—(1954) *Ann. N.Y. Acad. Sci.*, **60**, 251.
GROSS, L.—(1954) 'Leukaemia Research'. Ciba Foundation Symposium, edited by G. E. W. Walstenholme and M. P. Cameron. London (Churchill).
HEWITT, H. B.—(1953) *Brit. J. Cancer*, **7**, 367.—(1956) *Ibid.*, **10**, 564.
KLEIN, G. AND KLEIN, E.—(1956) *Ann. N.Y. Acad. Sci.*, **63**, 640.
MEYNELL, G. G.—(1957) *Biometrics*, **13**, 149.
MIDER, G. B.—(1957) *J. nat. Cancer Inst.*, **19**, 191.
REED, L. J. AND MUENCH, H.—(1938) *Amer. J. Hyg.*, **27**, 493.
REVESZ, L.—(1955) *J. nat. Cancer Inst.*, **15**, 1691.
SCOTT, O. C. A.—(1957) *Brit. J. Cancer*, **11**, 130.
SKIPPER, H. E., SCHABEL, F. M., BELL, M., THOMSON, J. R. AND JOHNSON, L.—(1957) *Cancer Res.*, **17**, 717.
YOFFEY, J. M. AND COURTICE, F. C.—(1956) 'Lymphatics, Lymph and Lymphoid Tissue'. London (Edward Arnold Ltd.).


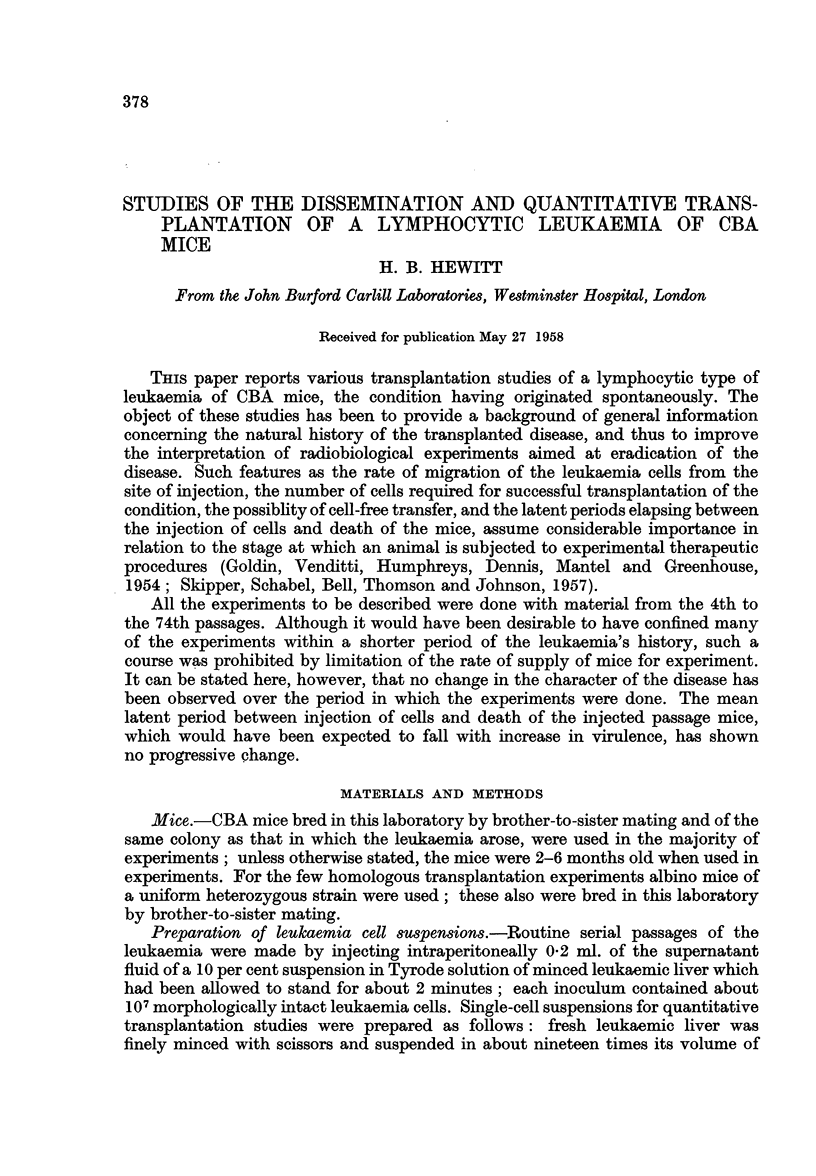

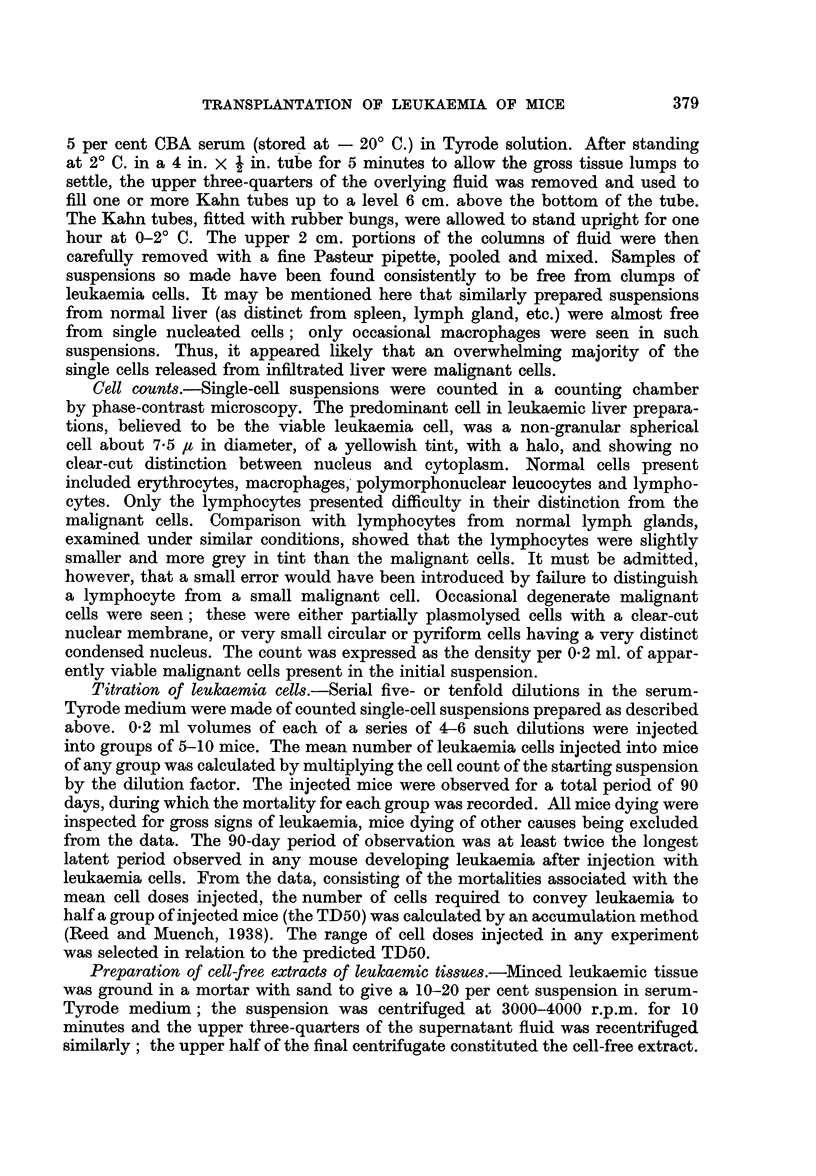

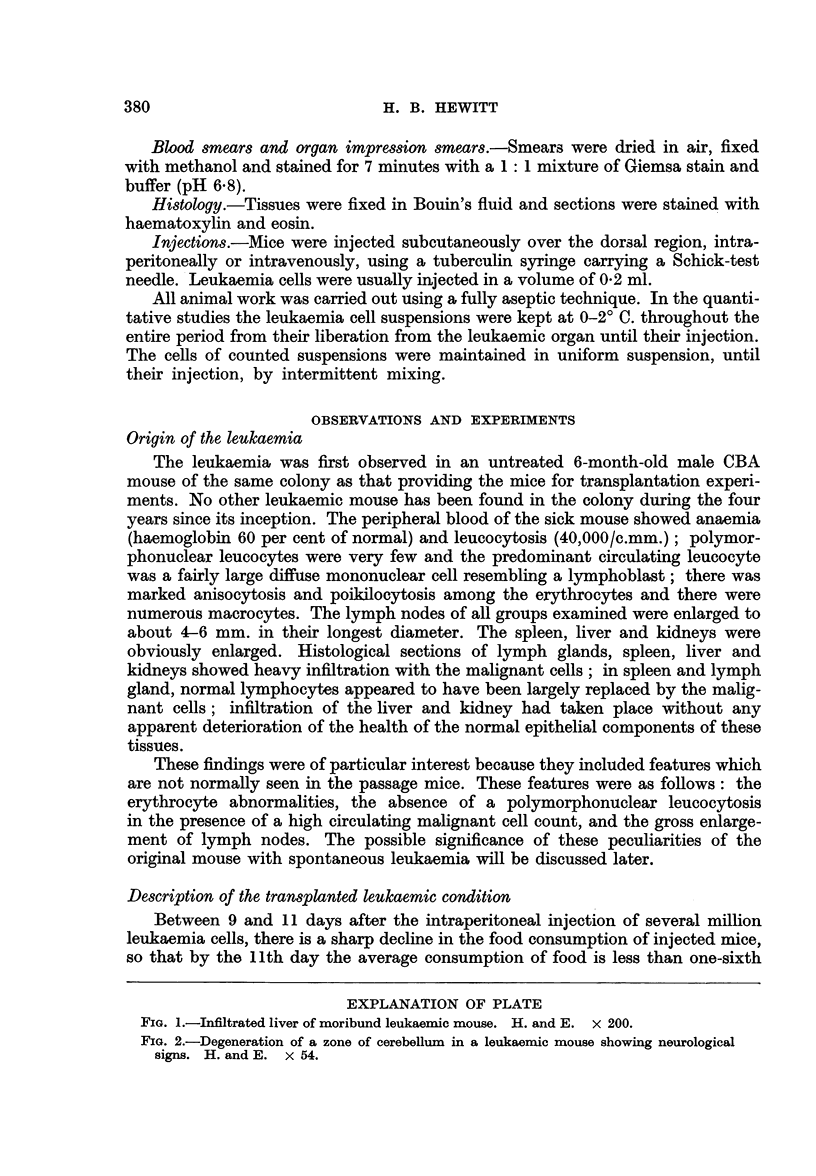

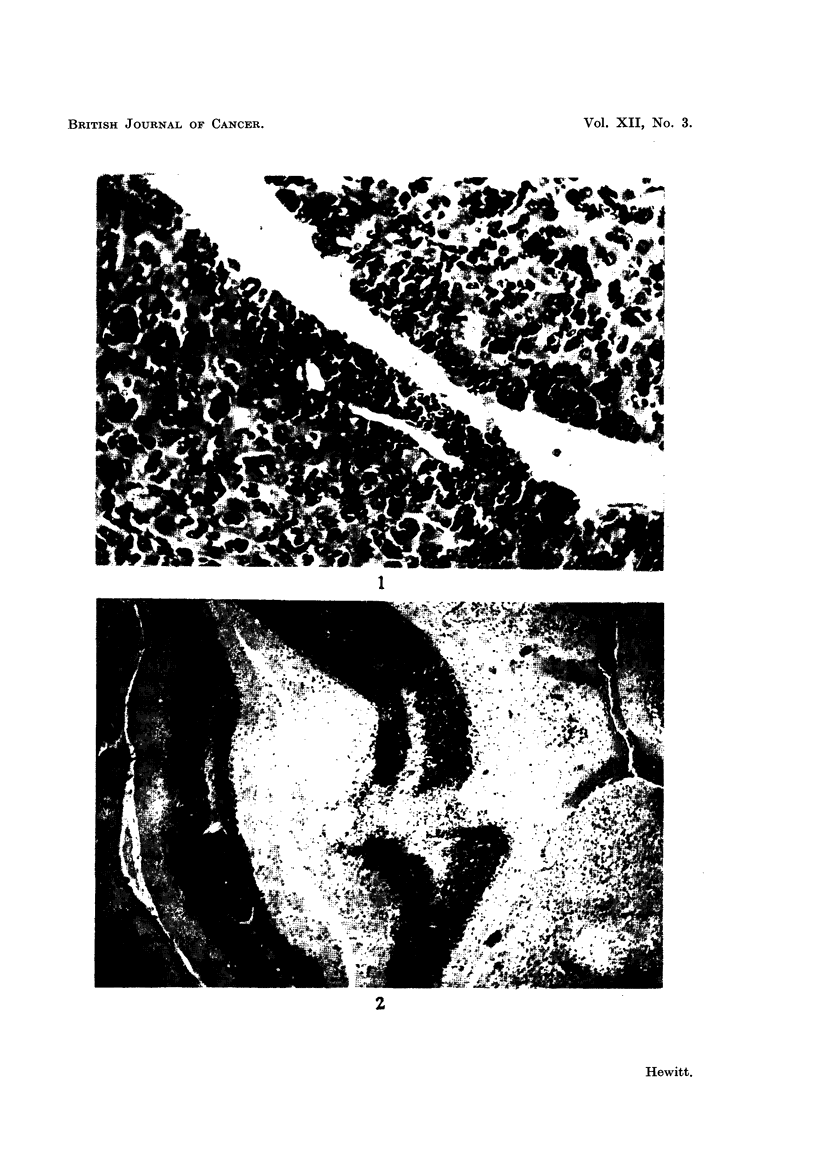

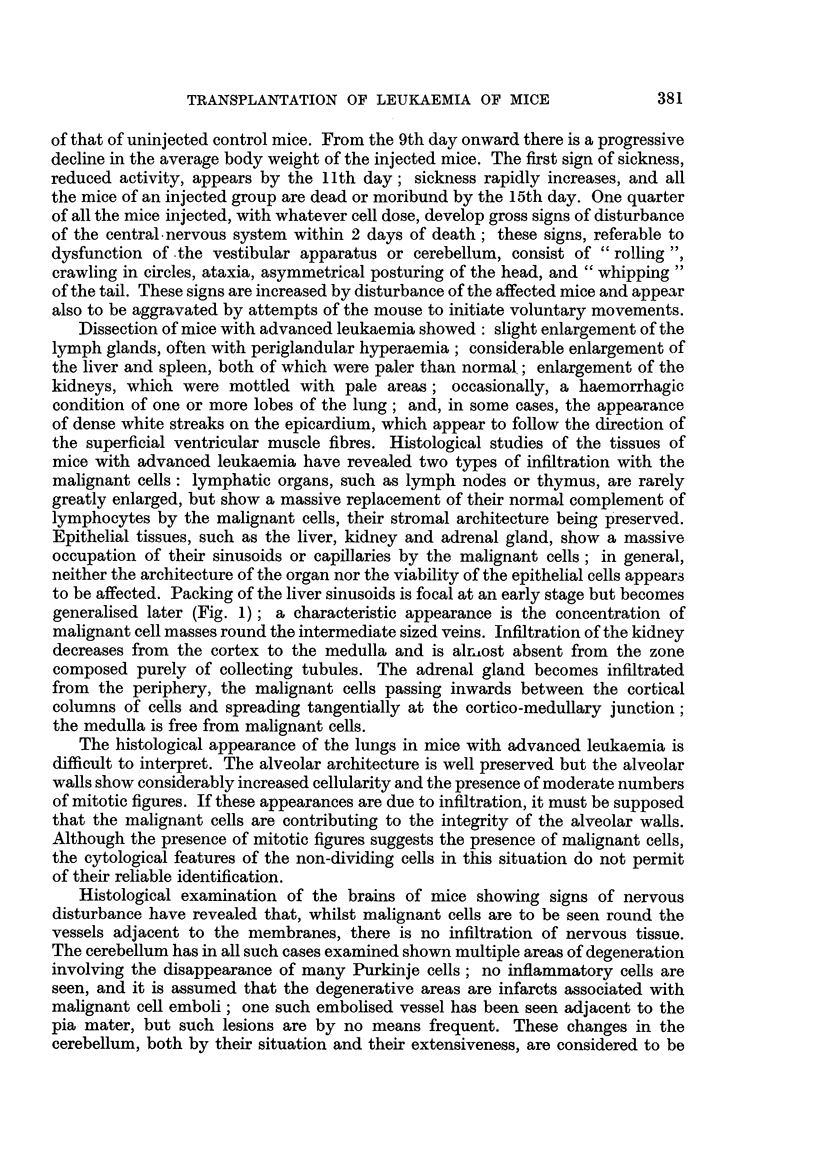

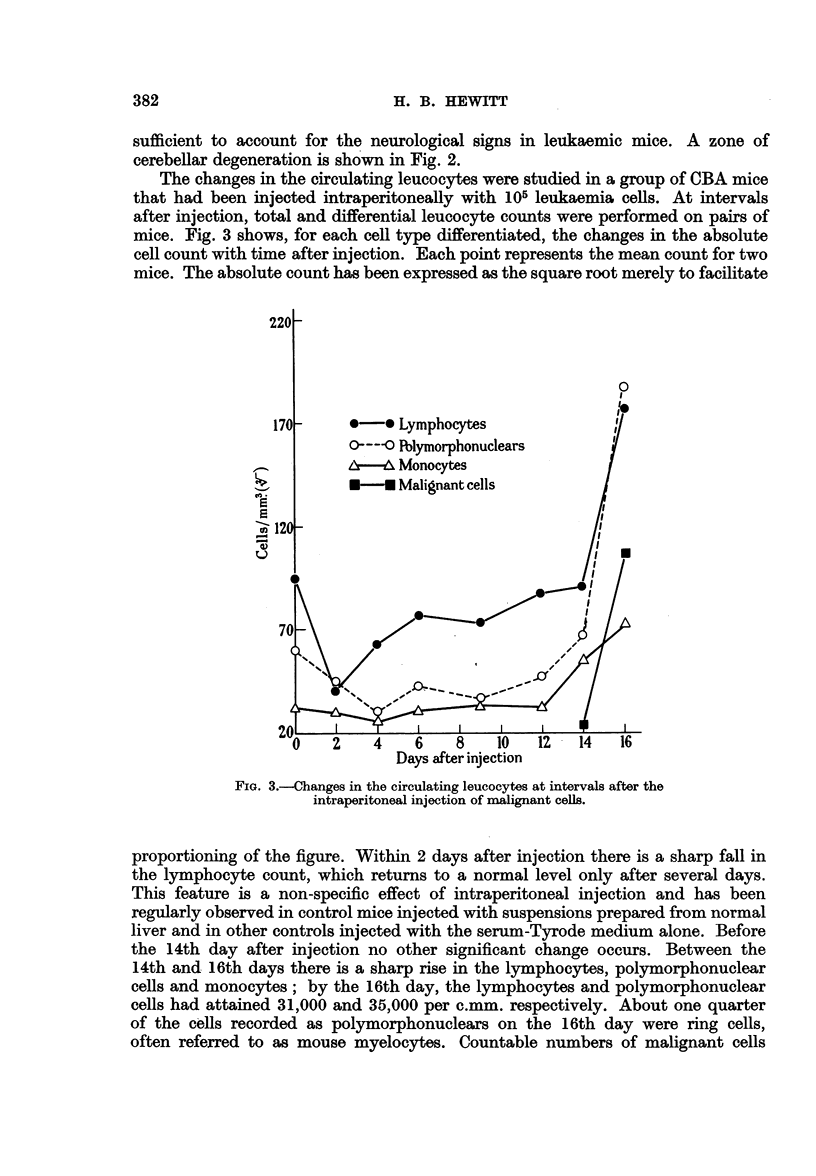

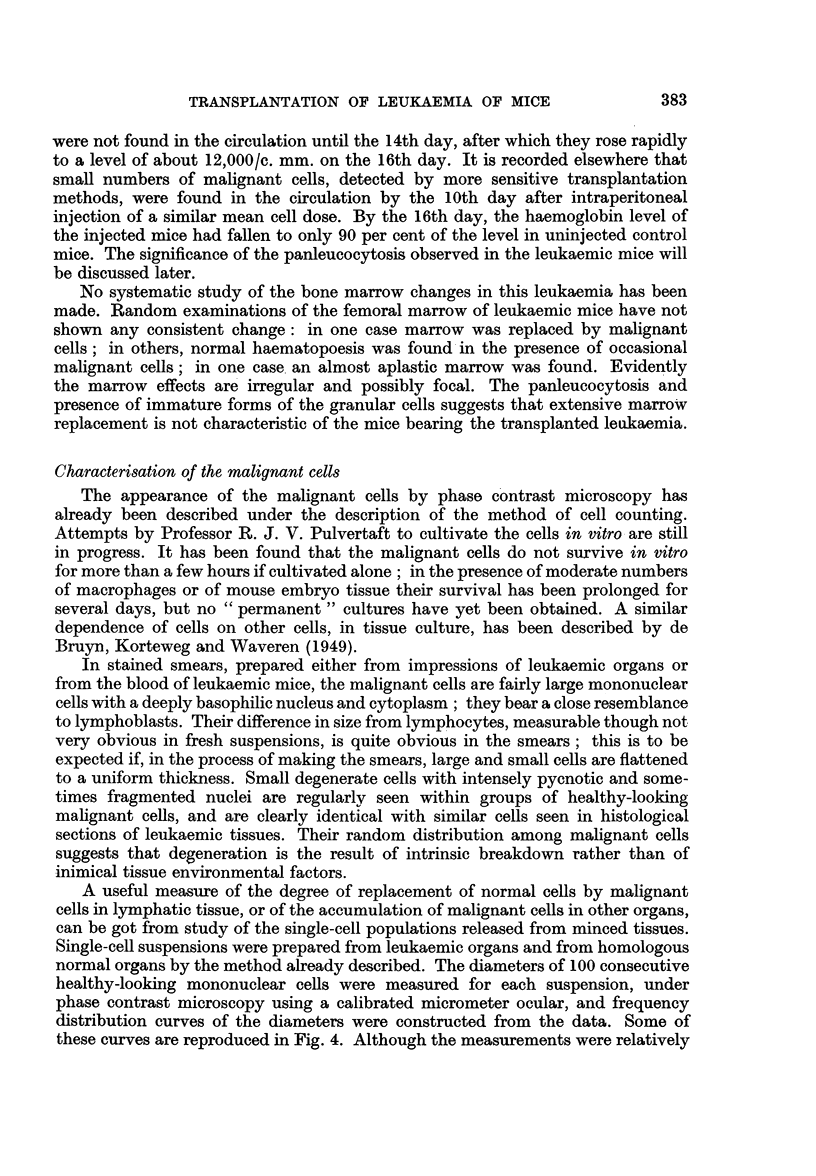

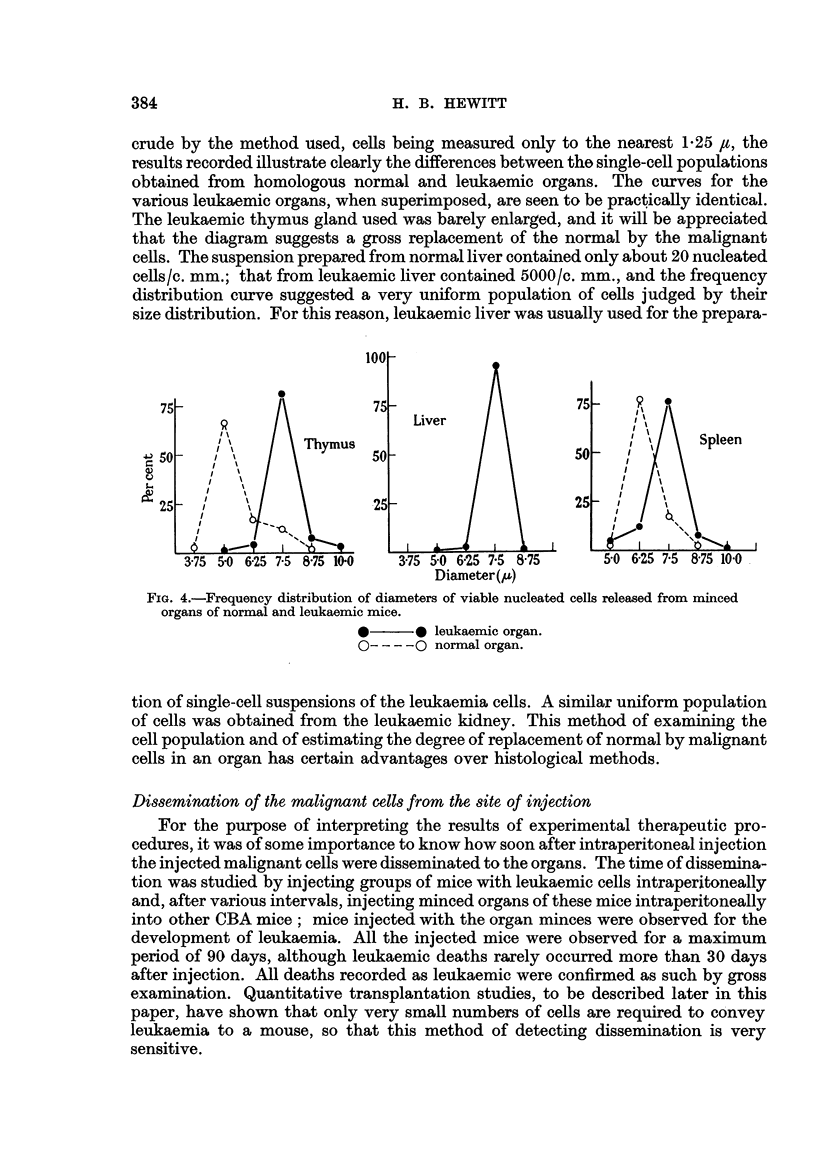

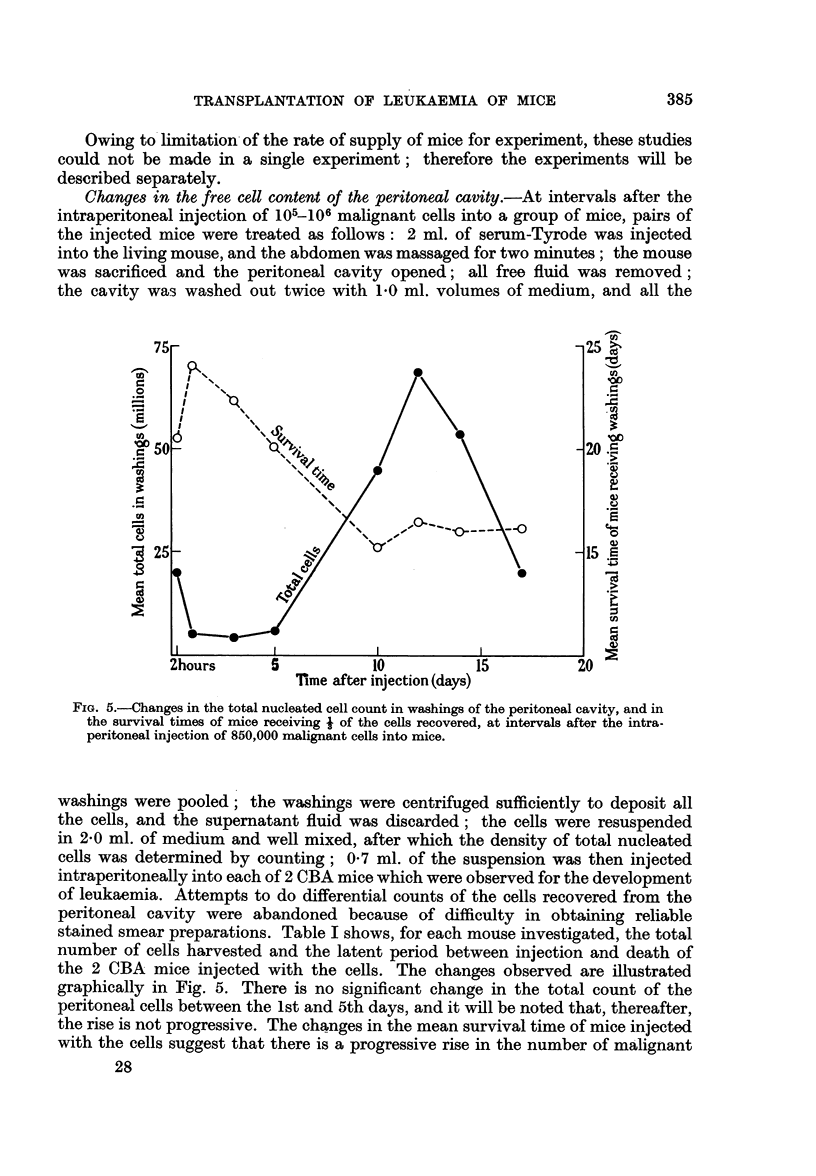

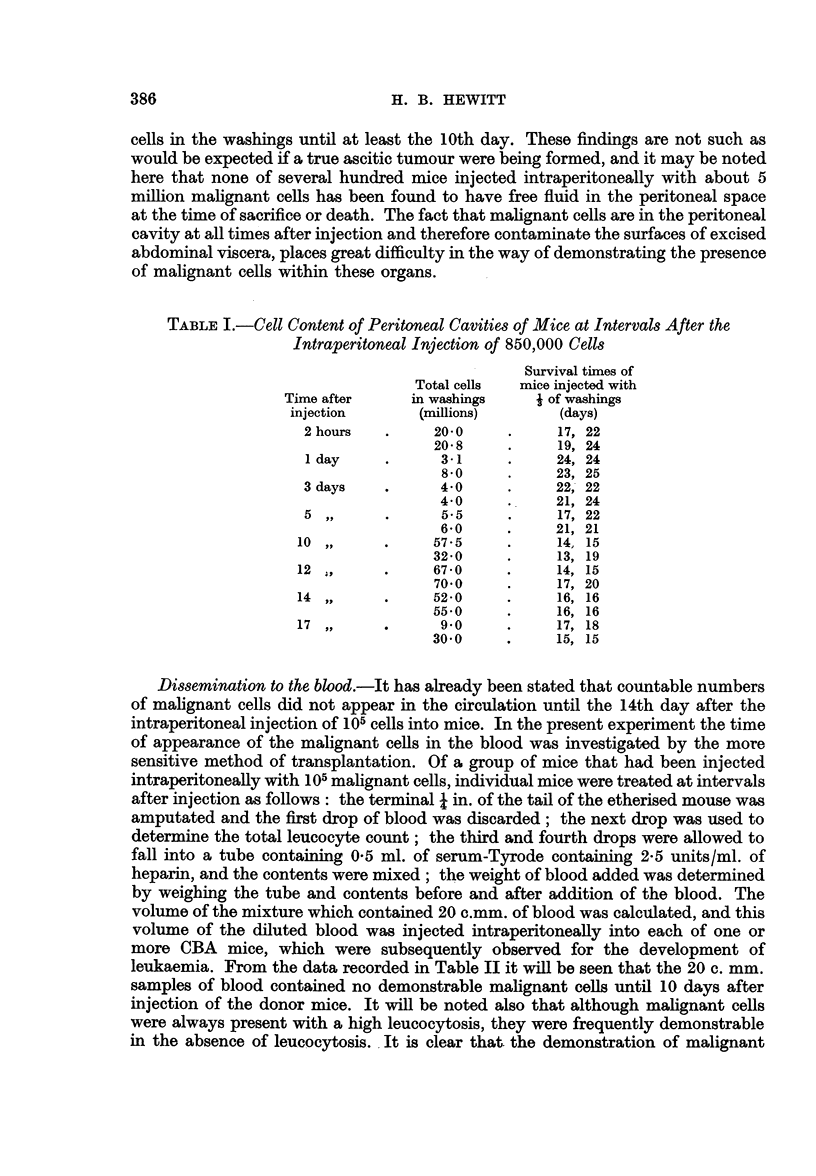

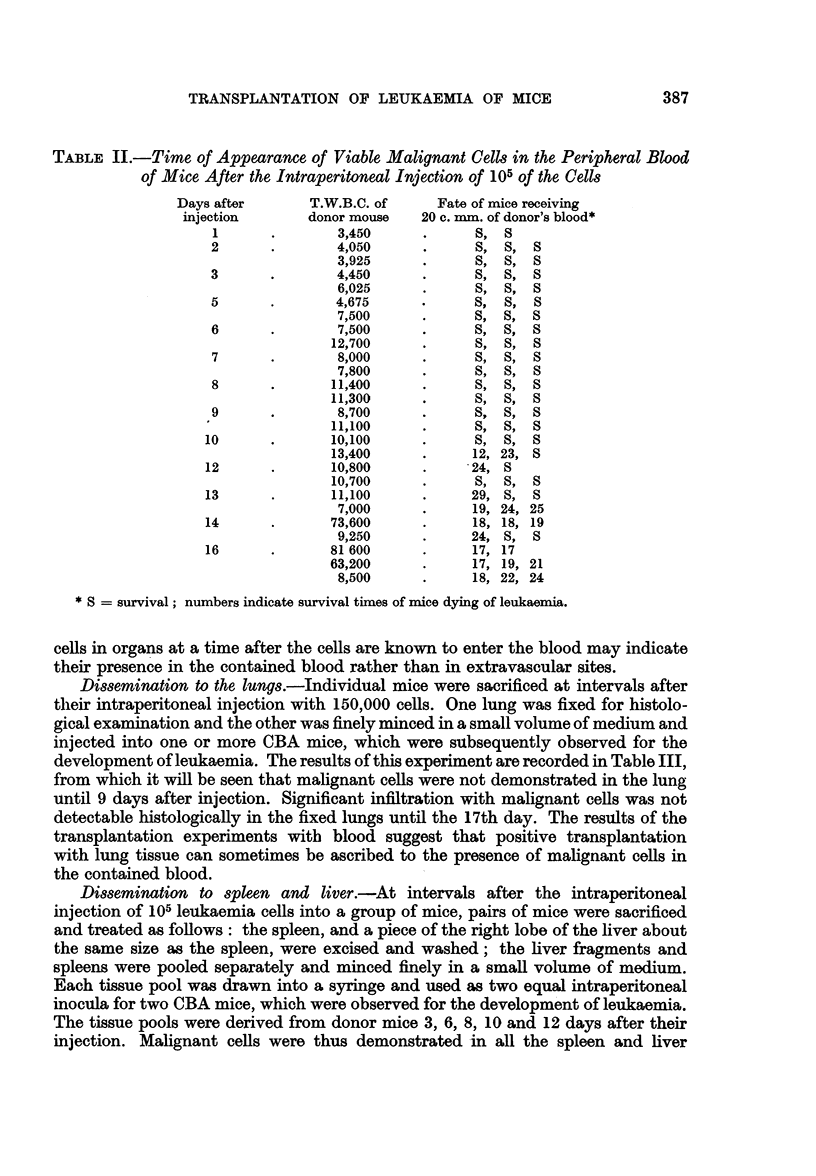

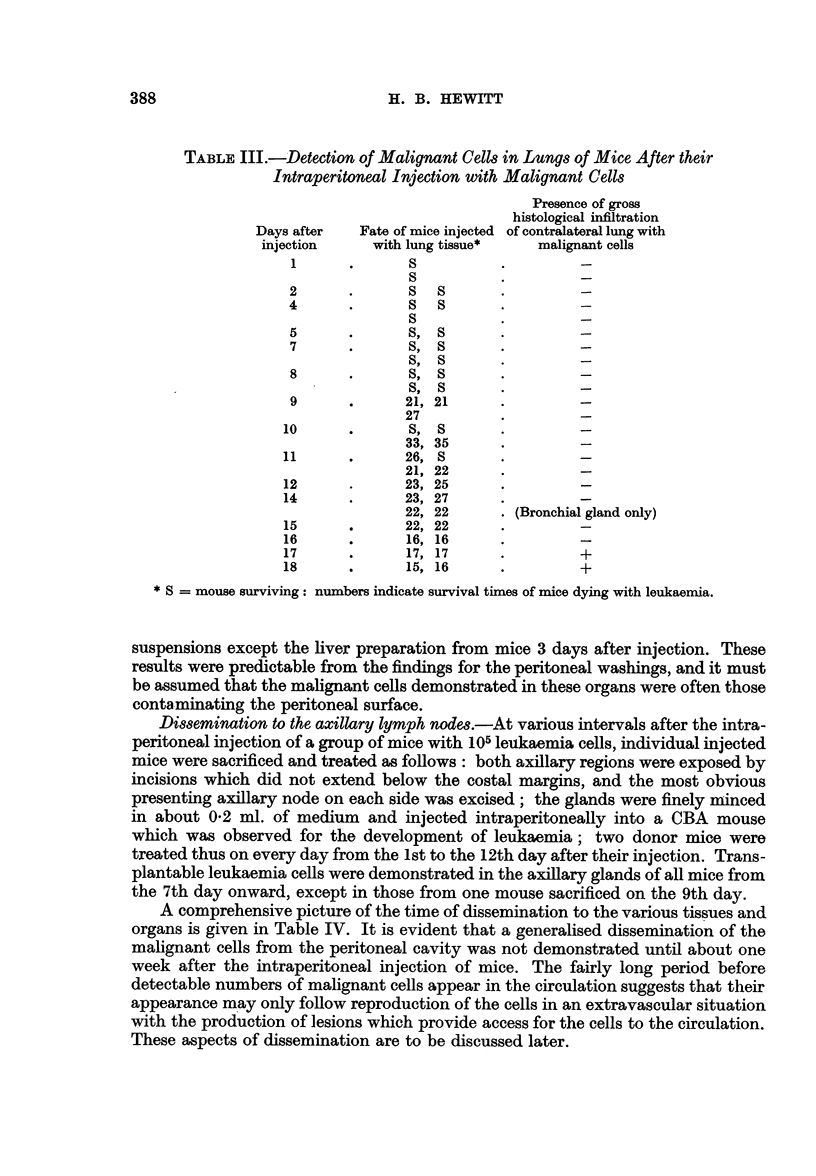

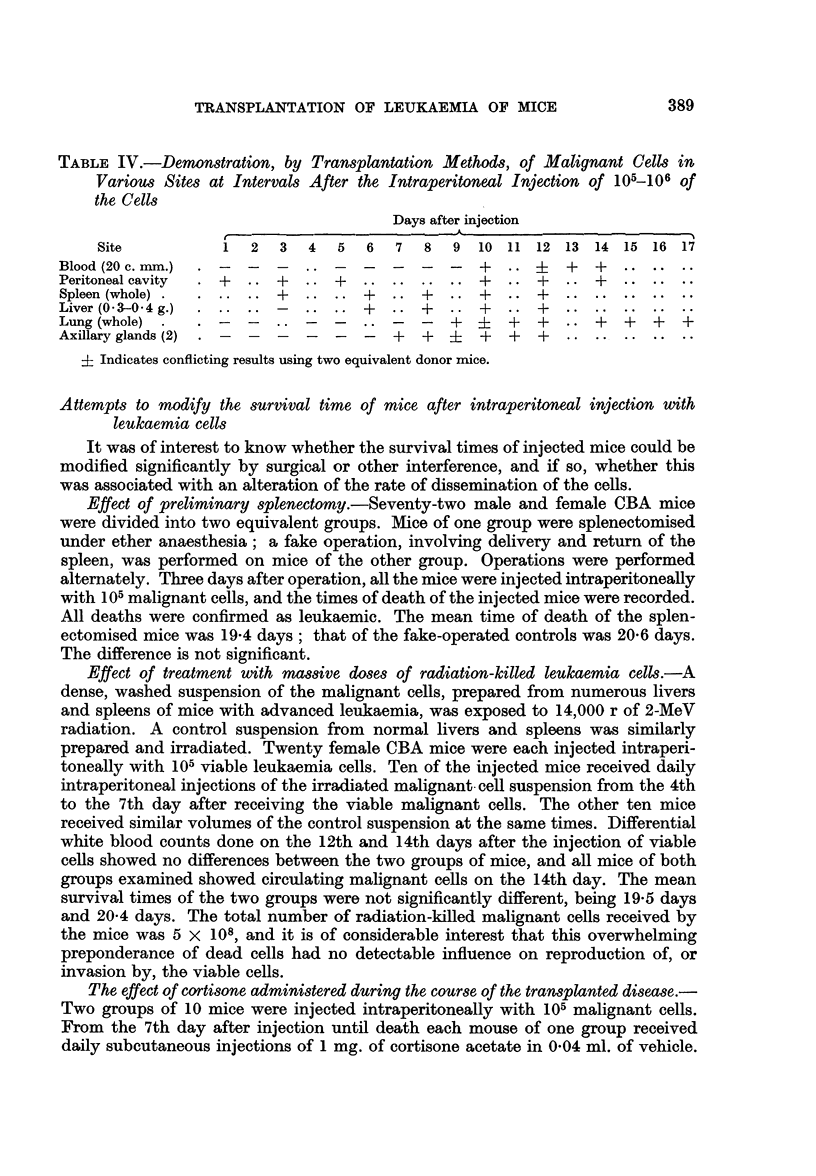

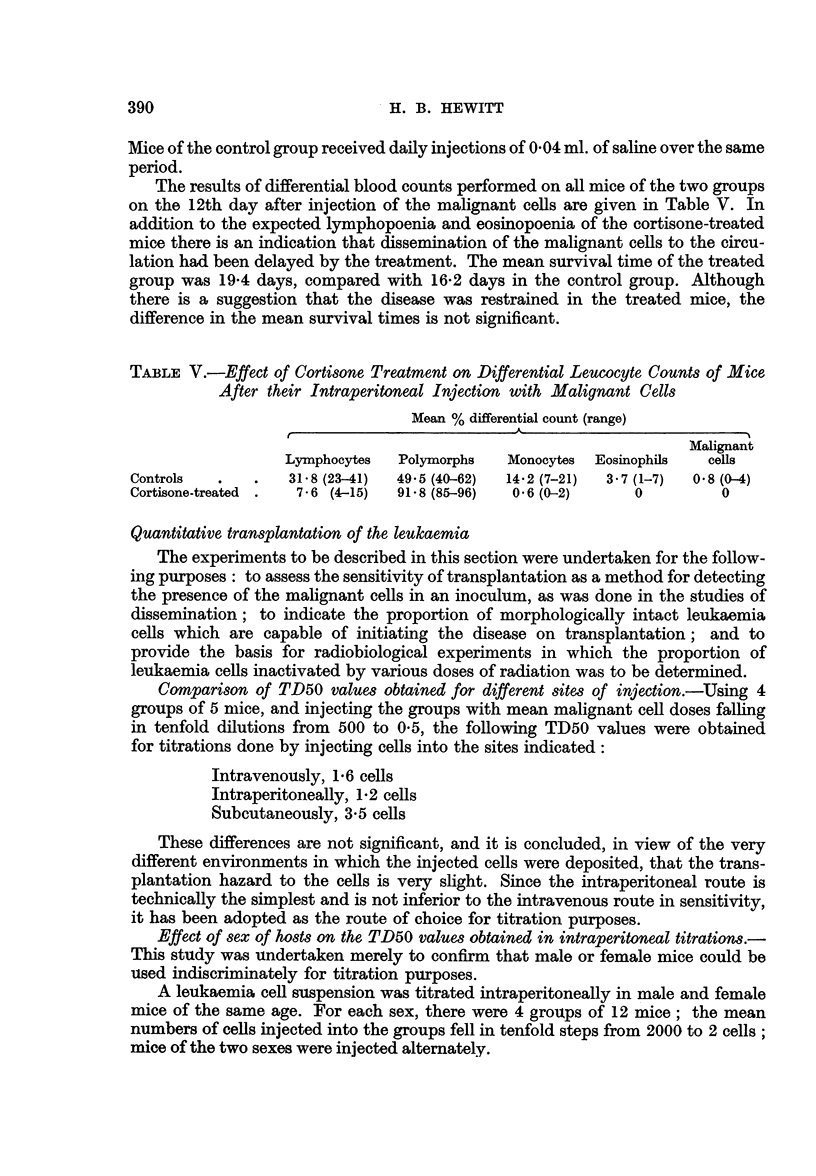

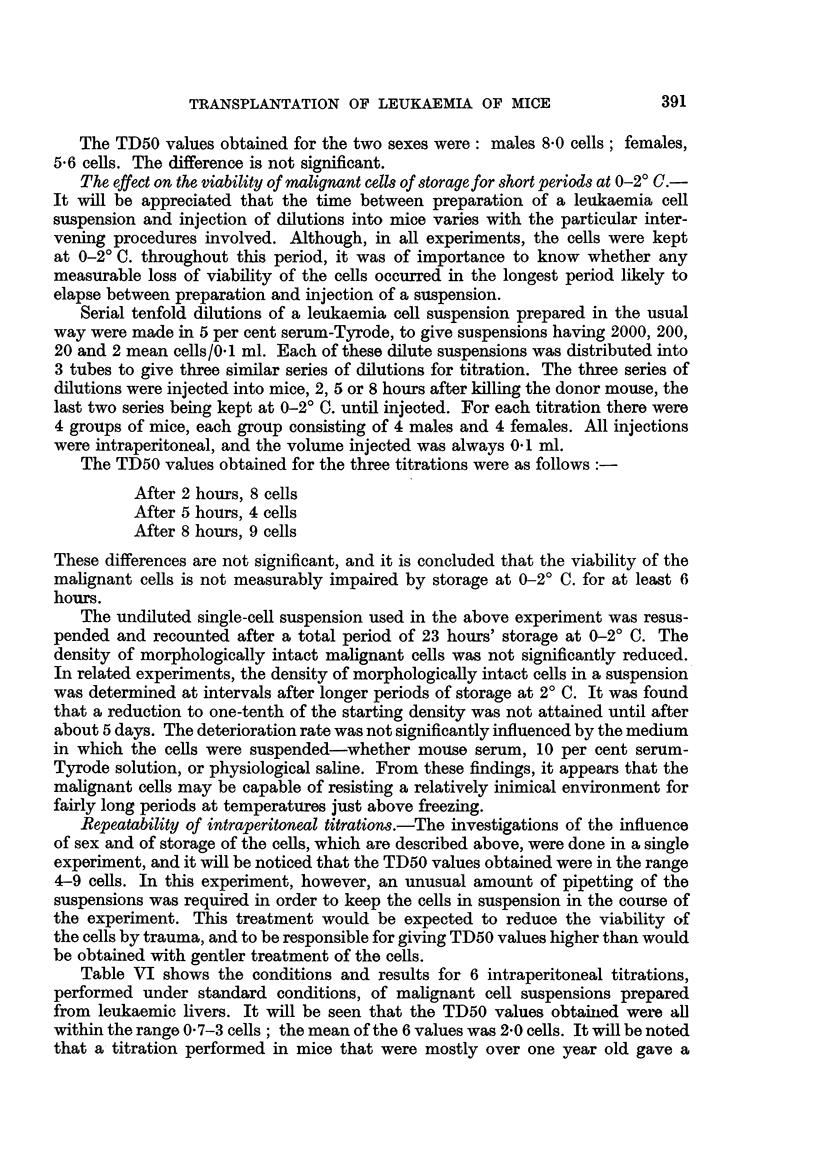

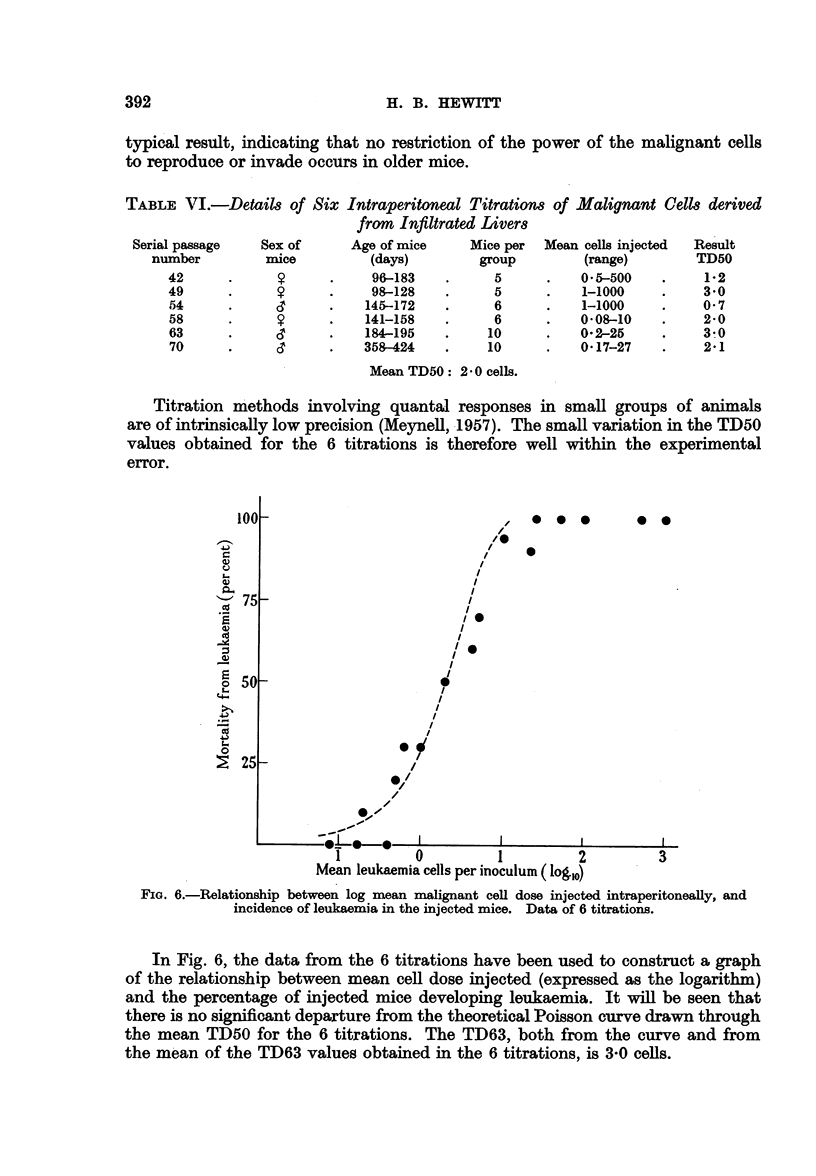

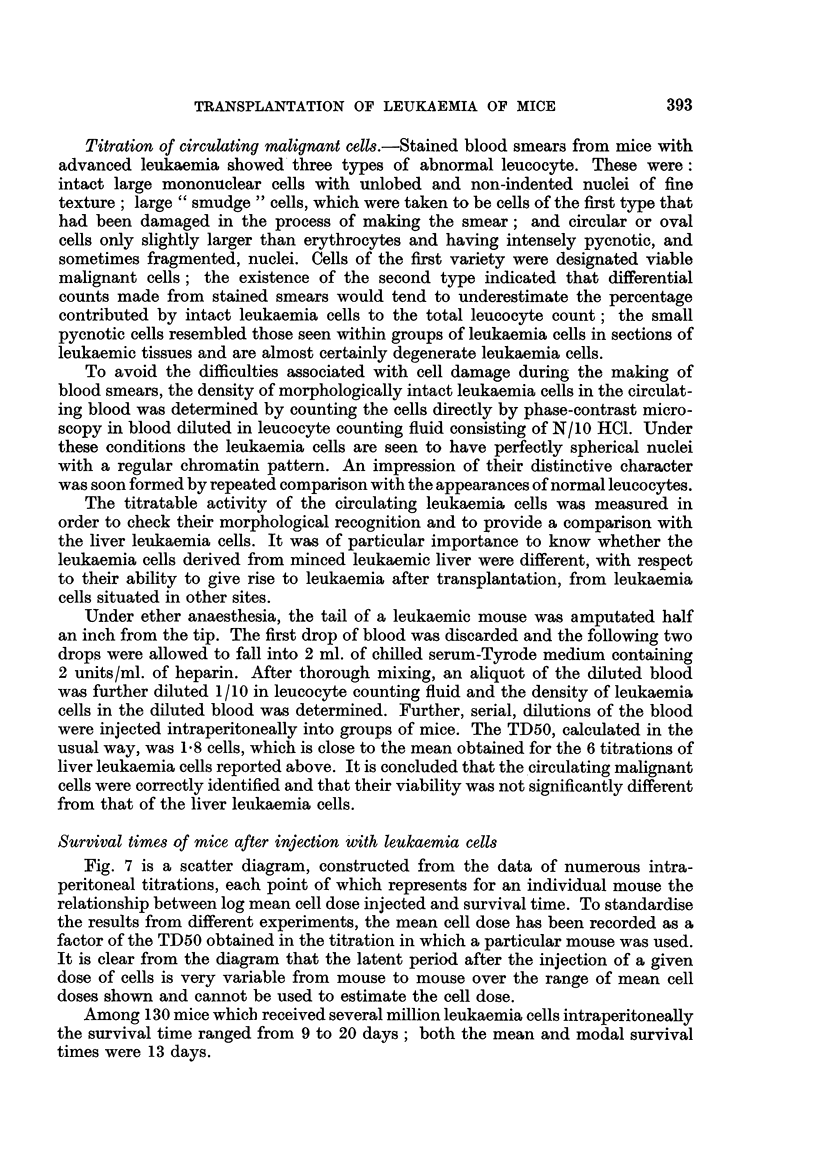

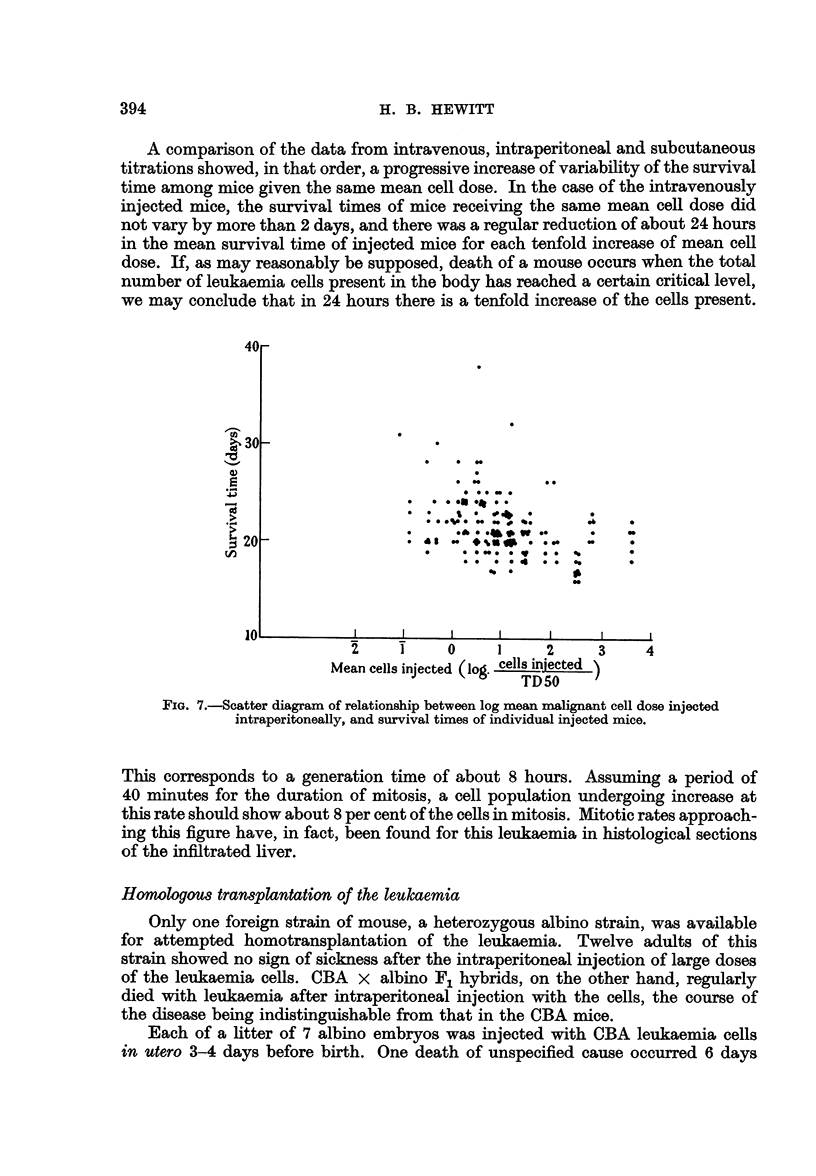

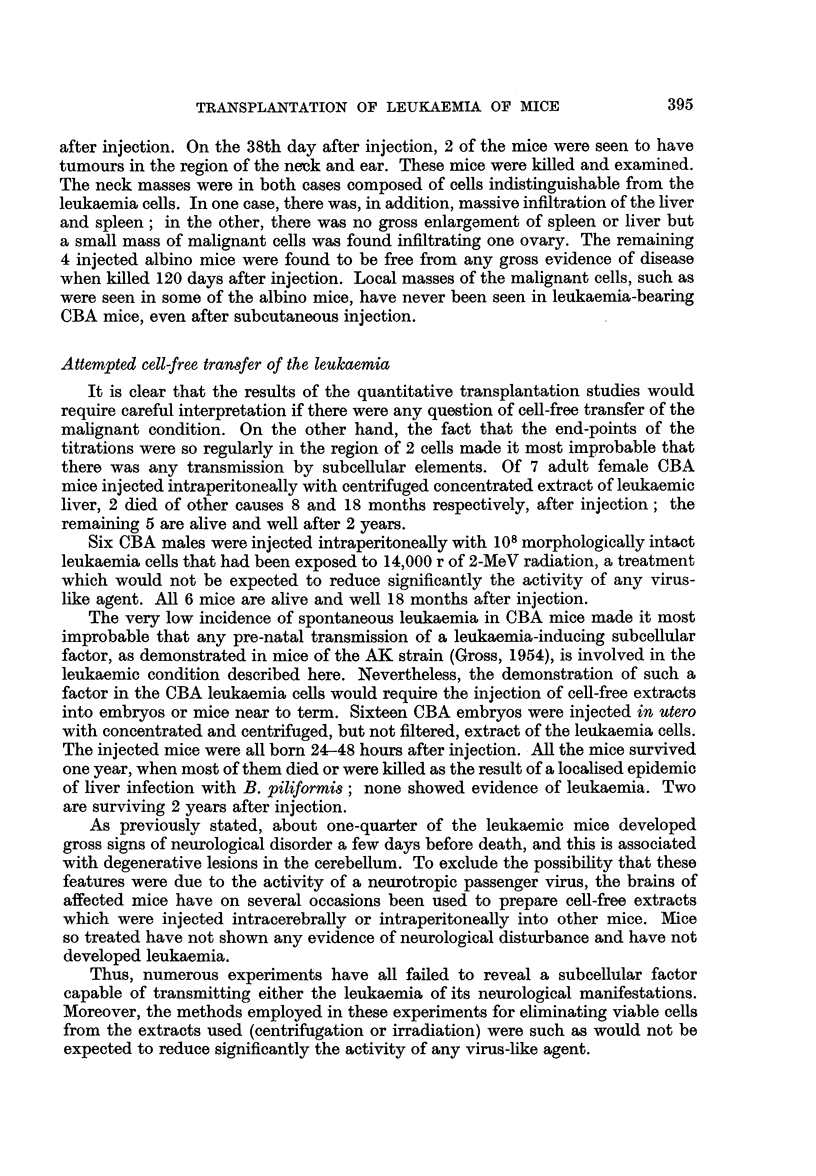

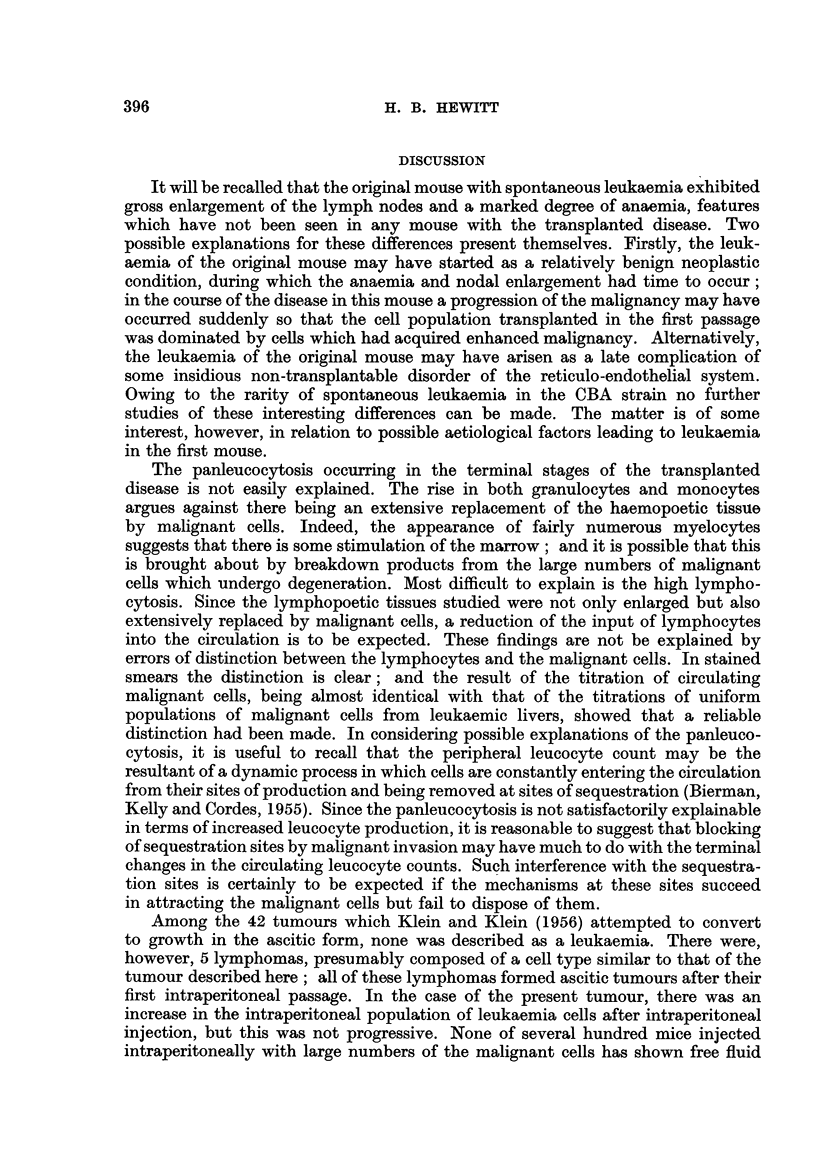

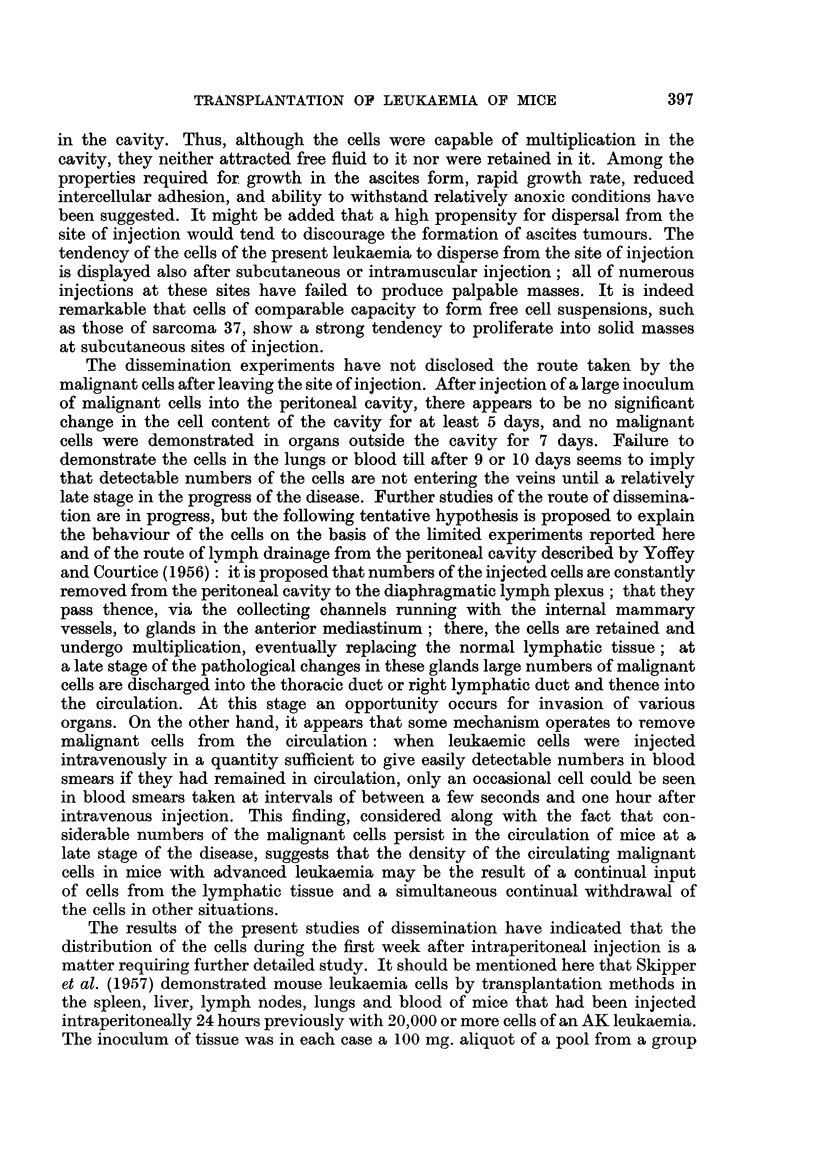

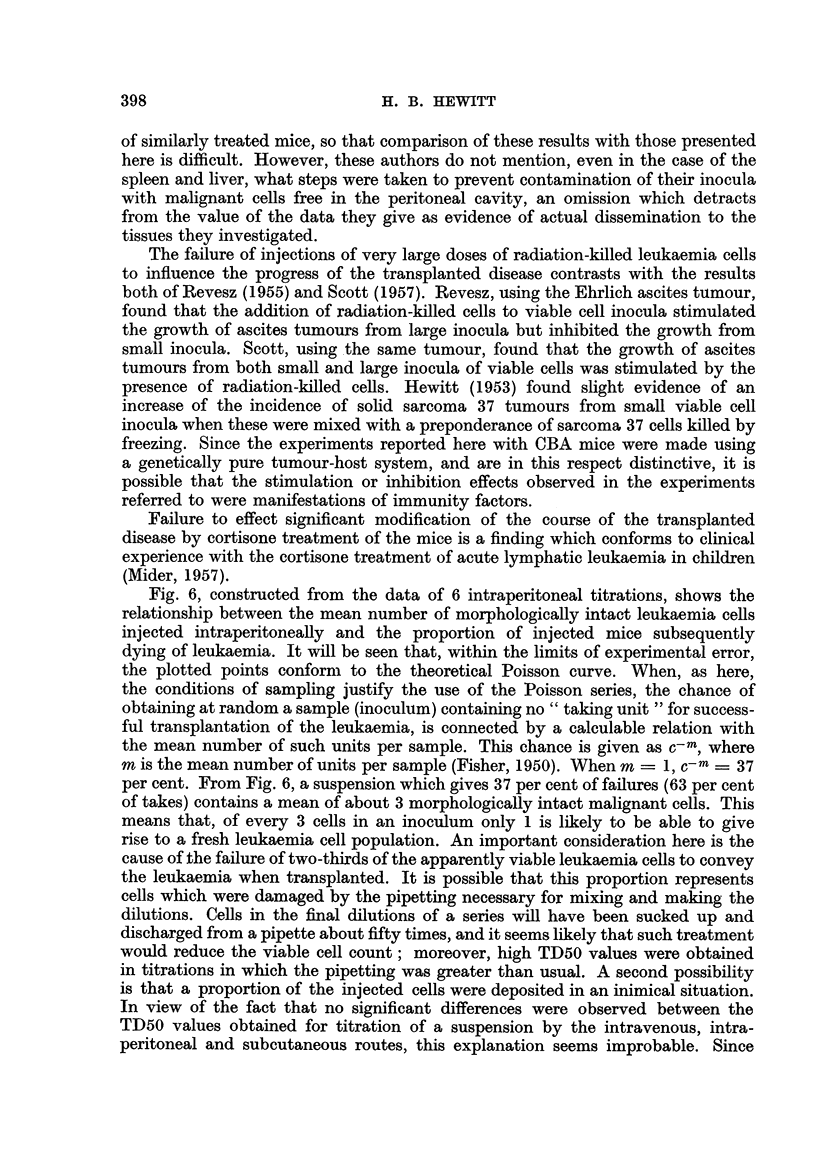

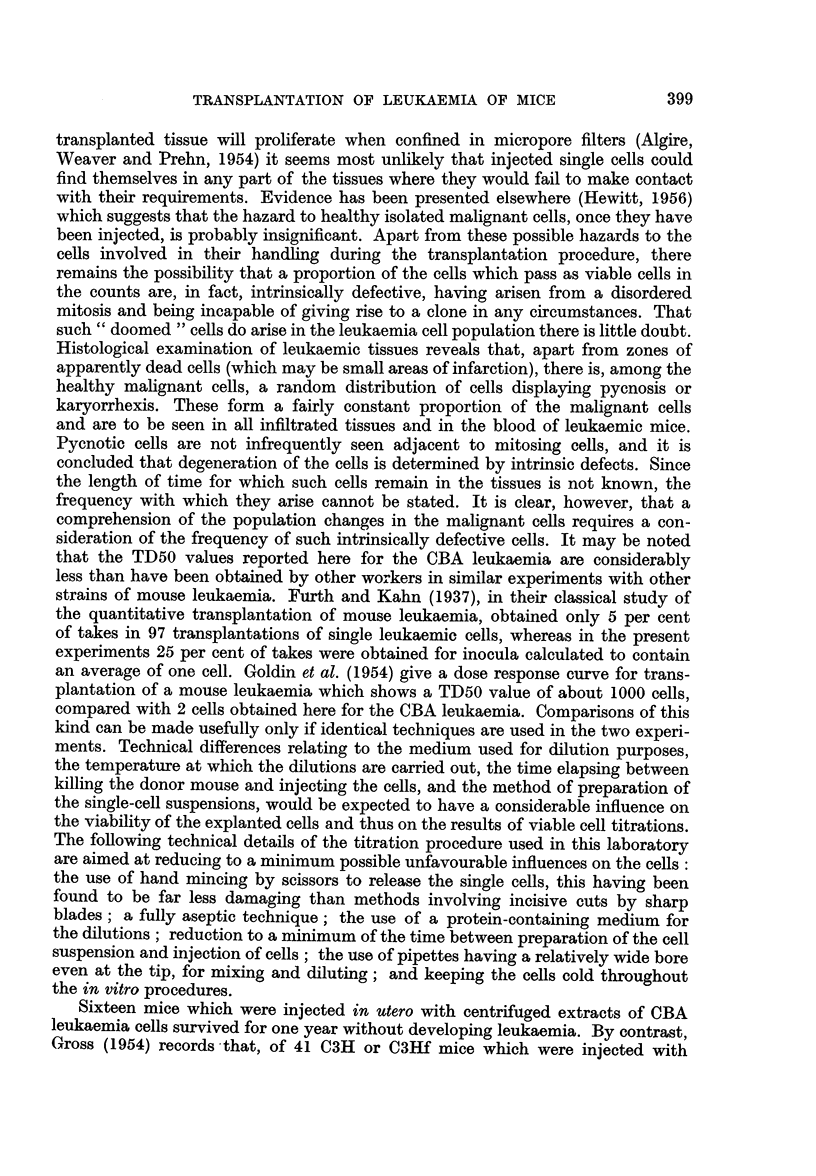

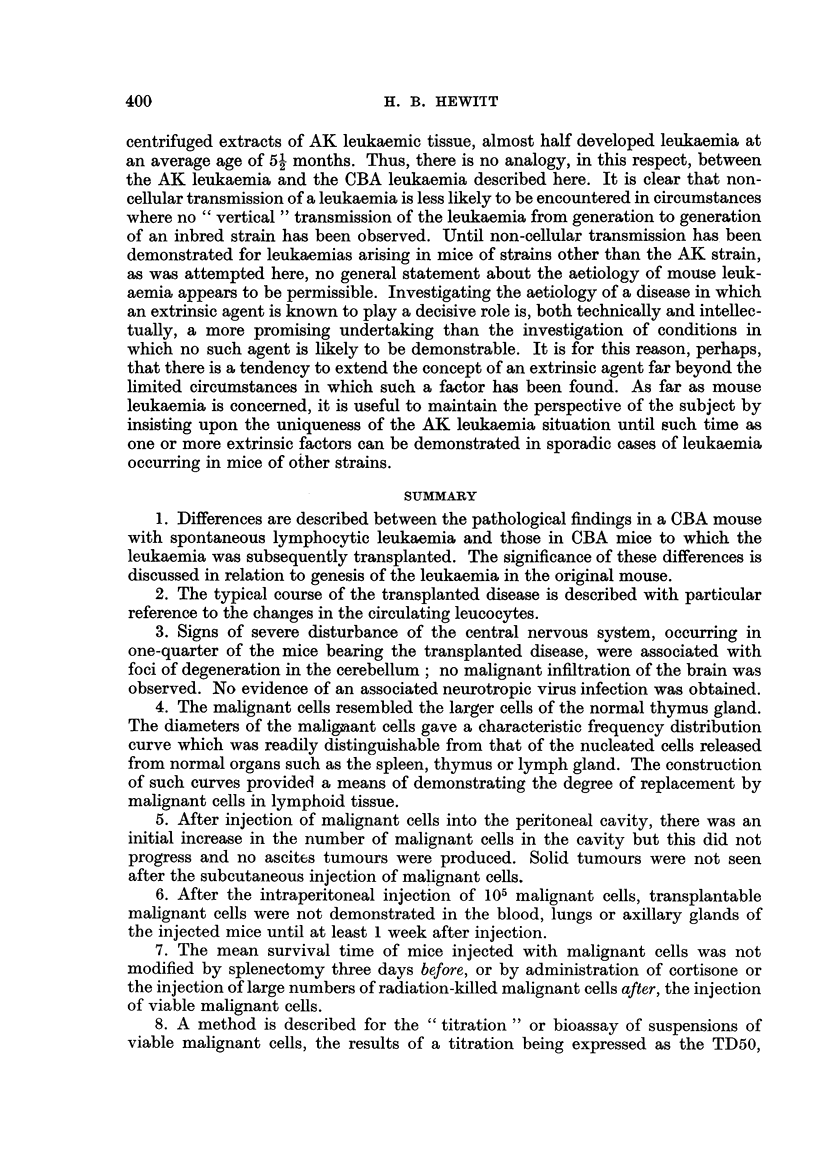

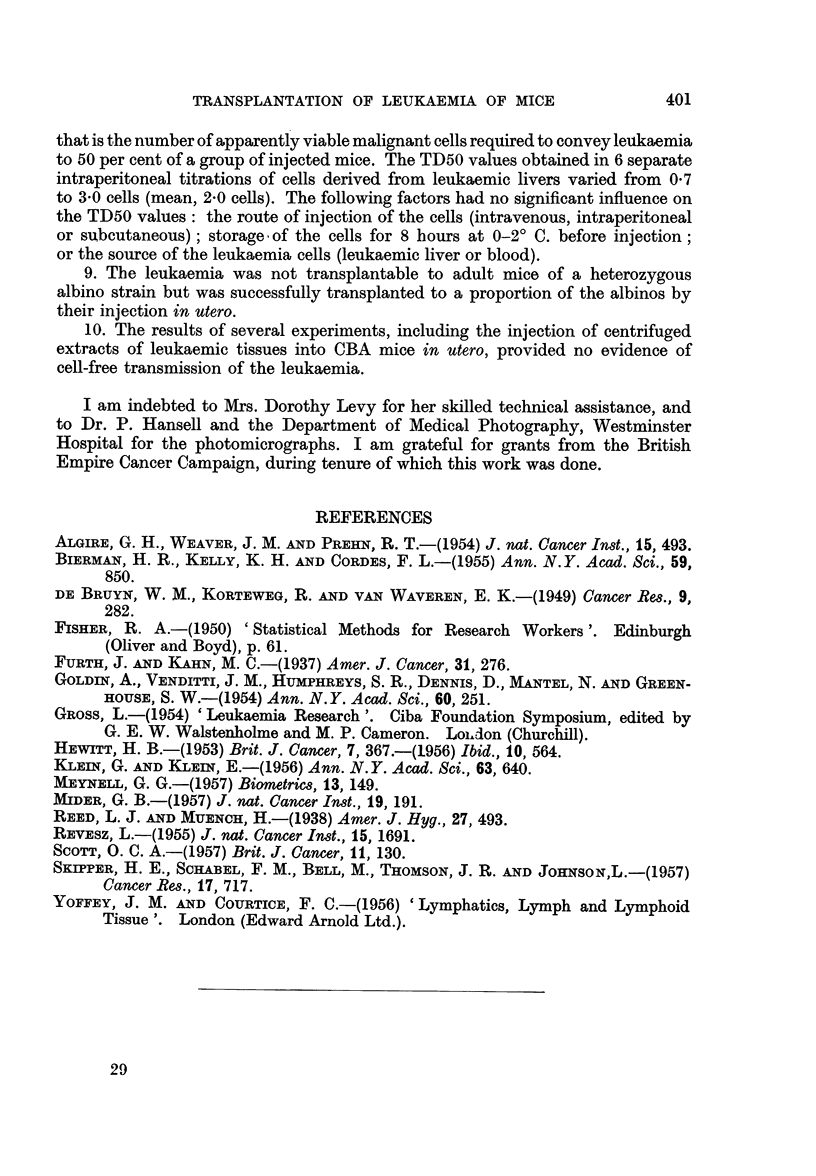


## References

[OCR_01507] ALGIRE G. H., WEAVER J. M., PREHN R. T. (1954). Growth of cells in vivo in diffusion chambers. I. Survival of homografts in immunized mice.. J Natl Cancer Inst.

[OCR_01523] GOLDIN A., VENDITTI J. M., HUMPHREYS S. R., DENNIS D., MANTEL N., GREENHOUSE S. W. (1954). Studies on the toxicity and antileukemic action of 6-mercaptopurine in mice.. Ann N Y Acad Sci.

[OCR_01530] KLEIN G., KLEIN E. (1956). Conversion of solid neoplasms into ascites tumors.. Ann N Y Acad Sci.

[OCR_01532] MIDER G. B. (1957). Research at the National Cancer Institute.. J Natl Cancer Inst.

[OCR_01535] REVESZ L. (1955). Effect of X-irradiation on the growth of the Ehrlich ascites tumor.. J Natl Cancer Inst.

[OCR_01538] SKIPPER H. E., SCHABEL F. M., BELL M., THOMSON J. R., JOHNSON S. (1957). On the curability of experimental neoplasms. I. Amethopterin and mouse leukemias.. Cancer Res.

